# c-JUN: a chromatin repressor that limits mesoderm differentiation in human pluripotent stem cells

**DOI:** 10.1093/nar/gkaf001

**Published:** 2025-01-29

**Authors:** Ran Zhang, Guihuan Li, Qi Zhang, Zhenhua Wang, Dan Xiang, Xiaofei Zhang, Jiekai Chen, Andrew P Hutchins, Dajiang Qin, Huanxing Su, Duanqing Pei, Dongwei Li

**Affiliations:** State Key Laboratory of Quality Research in Chinese Medicine, Institute of Chinese Medical Sciences, University of Macau, Avenida da Universidade, Taipa, Macao, 999078, China; Key Laboratory of Biological Targeting Diagnosis, Therapy and Rehabilitation of Guangdong Higher Education Institutes, The Fifth Affiliated Hospital of Guangzhou Medical University, 621 Gangwan Road, Huangpu District, Guangzhou, Guangdong, 510799, China; Key Laboratory of Biological Targeting Diagnosis, Therapy and Rehabilitation of Guangdong Higher Education Institutes, The Fifth Affiliated Hospital of Guangzhou Medical University, 621 Gangwan Road, Huangpu District, Guangzhou, Guangdong, 510799, China; Key Laboratory of Biological Targeting Diagnosis, Therapy and Rehabilitation of Guangdong Higher Education Institutes, The Fifth Affiliated Hospital of Guangzhou Medical University, 621 Gangwan Road, Huangpu District, Guangzhou, Guangdong, 510799, China; CAS Key Laboratory of Regenerative Biology, Guangdong Provincial Key Laboratory of Stem Cell and Regenerative Medicine, GIBH-HKU Guangdong-Hong Kong Stem Cell and Regenerative Medicine Research Centre, Hong Kong Institute of Science & Innovation, Guangzhou Institutes of Biomedicine and Health, 190 Kaiyuan Avenue, Science Park, Guangzhou, Guangdong 510530, China; CAS Key Laboratory of Regenerative Biology, Guangdong Provincial Key Laboratory of Stem Cell and Regenerative Medicine, GIBH-HKU Guangdong-Hong Kong Stem Cell and Regenerative Medicine Research Centre, Hong Kong Institute of Science & Innovation, Guangzhou Institutes of Biomedicine and Health, 190 Kaiyuan Avenue, Science Park, Guangzhou, Guangdong 510530, China; CAS Key Laboratory of Regenerative Biology, Guangdong Provincial Key Laboratory of Stem Cell and Regenerative Medicine, GIBH-HKU Guangdong-Hong Kong Stem Cell and Regenerative Medicine Research Centre, Hong Kong Institute of Science & Innovation, Guangzhou Institutes of Biomedicine and Health, 190 Kaiyuan Avenue, Science Park, Guangzhou, Guangdong 510530, China; Department of Systems Biology, School of Life Sciences, Southern University of Science and Technology, 1088 Xueyuan Blvd, Nanshan District, Shenzhen, 518055, China; Key Laboratory of Biological Targeting Diagnosis, Therapy and Rehabilitation of Guangdong Higher Education Institutes, The Fifth Affiliated Hospital of Guangzhou Medical University, 621 Gangwan Road, Huangpu District, Guangzhou, Guangdong, 510799, China; State Key Laboratory of Quality Research in Chinese Medicine, Institute of Chinese Medical Sciences, University of Macau, Avenida da Universidade, Taipa, Macao, 999078, China; Laboratory of Cell Fate Control, School of Life Sciences, Westlake University, No. 600 Dunyu Road, Xihu District, Hangzhou, 310024, China; Key Laboratory of Biological Targeting Diagnosis, Therapy and Rehabilitation of Guangdong Higher Education Institutes, The Fifth Affiliated Hospital of Guangzhou Medical University, 621 Gangwan Road, Huangpu District, Guangzhou, Guangdong, 510799, China; Hainan Provincial Key Laboratory for human reproductive medicine and Genetic Research&Key Laboratory of Reproductive Health Diseases Research and Translation, Ministry of Education, The First Affiliated Hospital of Hainan Medical University, Hainan Medical University, No. 3 Xueyuan Road, Longhua District, Haikou, Hainan, 571101, China

## Abstract

Cell fate determination at the chromatin level is not fully comprehended. Here, we report that *c-JUN* acts on chromatin loci to limit mesoderm cell fate specification as cells exit pluripotency. Although *c-JUN* is widely expressed across various cell types in early embryogenesis, it is not essential for maintaining pluripotency. Instead, it functions as a repressor to constrain mesoderm development while having a negligible impact on ectoderm differentiation. c-JUN interacts with MBD3–*NuRD* complex, which helps maintain chromatin in a low accessibility state at mesoderm-related genes during the differentiation of human pluripotent stem cells into mesoderm. Furthermore, *c-JUN* specifically inhibits the activation of key mesoderm factors, such as *EOMES* and *GATA4*. Knocking out *c-JUN* or inhibiting it with a JNK inhibitor can alleviate this suppression, promoting mesoderm cell differentiation. Consistently, knockdown of *MBD3* enhances mesoderm generation, whereas *MBD3* overexpression impedes it. Overexpressing *c-JUN* redirects differentiation toward a fibroblast-like lineage. Collectively, our findings suggest that *c-JUN* acts as a chromatin regulator to restrict the mesoderm cell fate.

## Graphical Abstract

**Graphical Abstract:**
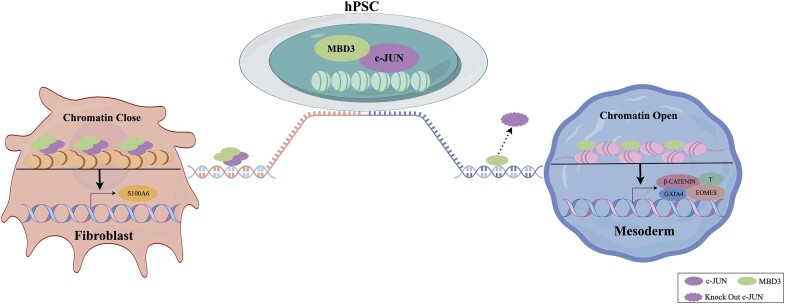

## Introduction

Human early embryonic development, especially peri-implantation, is highly dynamic but also difficult to study due to a lack of access for research (1,2). Thus, *in vitro* models are valuable for deciphering the processes of cellular differentiation. Human pluripotent stem cells (hPSCs) serve as an excellent model for this purpose, as they can differentiate into all three germ layers *in vitro* and provide an opportunity to study the intricate mechanism of lineage commitment (3). During gastrulation, signaling pathways, including WNT, BMP and Nodal, are instrumental in regulating mesoderm fate determination (4–9). These pathways initiate a cascade of molecular events, activating specific transcription factors (TFs) such as *T* (*BRACHYURY*), *EOMES*, *GATA4* and *MESP1*, which are essential for mesoderm development (10–13), and further development into diverse tissues and organs such as muscles, bones, blood vessels and the cardiovascular system in late embryonic development (14,15).

Cell fate decisions are crucial in development (16,17), and *c-JUN*, a TF belonging to the activator protein 1 (AP-1) family (18), plays a significant role in these processes (19,20). In mice, *c-JUN* deficiency results in embryonic lethality around E13.5 (21,22), and it also suppresses fibroblast proliferation and disrupts the cell cycle (23–25). *c-JUN* impedes the reprogramming of Mouse Embryonic Fibroblasts (MEFs) into induced pluripotent stem cells (iPSCs), indicating its importance in cellular reprogramming and plasticity (23,26). In humans, knocking out *c-JUN* or inhibiting its activity enhances definitive endoderm differentiation (27). Our previous study showed that knocking out *c-JUN* promotes the differentiation of hPSCs into cardiomyocytes, which is a mesoderm-derived cell type, while *c-JUN* overexpression hinders this process (28). Additionally, a study reported that knocking out *Stk40* elevates c-JUN protein levels, thereby impairing mesoderm cell fate decisions (29). However, the precise mechanism of how *c-JUN* regulates mesoderm cell fate is not yet fully understood.

Here, we reveal that c-JUN can maintain the closed state of mesoderm chromatin thereby inhibiting the differentiation of hPSCs into mesoderm. Despite its wide expression in early embryogenesis, we found that endoderm and mesoderm marker genes were activated upon *c-JUN* knockout. Genomic analyses showed that c-JUN interacted with MBD3, one component of the repressor *NuRD* complex that regulate chromatin remodeling and gene repression, which in turn suppressed the transcriptional activation of key mesoderm-associated genes such as *HAND1, EOMES* and *GATA4*. Knockout c-JUN or knockdown MBD3 facilitates mesoderm differentiation. Notably, *c-JUN* overexpression redirects cell differentiation toward a fibroblast-like lineage. Overall, our results highlight the inhibitory role of *c-JUN* in the commitment to a mesodermal cell fate during early human embryonic development.

## Materials and methods

### Cell culture

The hPSCs (H1) (WiCell, WA01) and all genetically modified cell lines were plated on Matrigel-coated (Corning, 354234) wells in mTeSR1 (STEMCELL, 85850) medium containing 10 μM Y27632 (Selleckchem, S1049). HEK293T (ATCC, CRL-1126) cell was cultured in Dulbecco’s modified Eagle’s medium (DMEM) (Gibco) containing 10% fetal bovine serum (FBS) (ABW, AB-FBS0500), GlutaMAX (Gibco, 35050061) and MEM NEAA (Gibco, 11140050). All cell lines were cultured in an incubator at 37°C with 5% CO_2_ and were tested for mycoplasma using the Mycoalert™ mycoplasma detection kit (Lonza, LT07-318).

### Plasmid construction and cell line generation

The full-length encoding genes were amplified using the Phanta Max Super‐Fidelity DNA Polymerase kit (Vazyme, P505) and generated the clone plasmid using infusion cloning (Vazyme, C112-01). The human gene *c-JUN* was inserted into the PB vector for generating the expression plasmid PB-TRE3G*-c-JUN*-SV40 polyA. The clones for these genes: *c-JUN/MBD3/MTA2/MTA3* were inserted into the PB-CAG-bGH polyA vector. The single guide RNA (sgRNA) plasmid was constructed using the PX330 vector. The short hairpin RNA (shRNA) plasmid for *MBD3* gene was constructed using the pLKO.1-TRC cloning vector. The primer sequences for all cloned genes were provided in supplementary table.

Cell lines were obtained through electro-transfection using the Human Stem Cell Nucleofector® Starter Kit (Lonza, VPH-5002). The dissociated single hPSCs cells were resuspended with 100 μl of Human Stem Cell Nucleofector Kit, and then transfected using Lonza Nucleofector 2b, with program B016. Subsequently, 2.5 μg of each plasmid was used to electro-transfect into 1 × 10^6^ cells. The electro-transfected cells were seeded on Matrigel-coated wells in mTeSR1 medium supplemented with 10 μM Y27632.

For the *c-JUN*-/- hPSCs, knockout cell line was generated through CRISPR/Cas9 (PX330 Vector), utilizing pairs of sgRNA to delete the target exons. Puromycin was used to select the cells. After ∼2 weeks, single clone was picked and expanded. To detect gene deletion, the individual clone genomic DNA was extracted and analyzed by polymerase chain reaction (PCR) using agarose gel electrophoresis. The positively identified product was further validated by Sanger sequencing, western blotting and karyotype analysis. The sgRNA sequences of *c-JUN* were as follows: sgRNA1: acaagtttcggggccgcaac; sgRNA2: gagaacttgacaagttgcga, as described in our previous research (28).

For over-expressing cell line, the cell line was generated by introducing PB-TRE3G*-c-JUN*-SV40 polyA in the *c-JUN*-/- cell line, for inducing the *c-JUN* gene expression by adding doxycycline (dox) (Selleckchem, S5159) to the culture medium. The other over-expressing cell lines were generated by introducing PB-CAG-*MBD3*-HA in hPSCs (H1).

### Differentiation of hPSCs into different germ layers

The chemically defined methodology used in this experiment followed the previous study (3). H1 cells were dissociated into individual cells and seeded at a density of 8 × 10^5^ cells on Matrigel-coated 12-well plates. They were initially cultured in mTeSR1 medium for 2 days before undergoing the following differentiation.

For inducing cell differentiating toward mesoderm, the cells were cultured for 1 day in RPMI/1640 (Gibco, C11875500BT) medium supplemented with 100 ng/ml Activin A (PeproTech, 120–14E), 10 ng/ml bFGF (PeproTech, 100–18B), 100 ng/ml BMP4 (R&D systems, 314-BP), 100 ng/ml VEGF (PeproTech, 100–20), 0.5% FBS (Gibco, 10091130), 200 mM GlutaMAX, 0.2× MEM non-essential amino acids solution and 55 μM of 2-mercaptoethanol. Subsequently, the cells were maintained in the same medium without Activin A for an additional 4 days. At day 5, the cells were collected for real-time reverse transcriptase-polymerase chain reaction (RT-PCR) analysis and total RNA sequencing (RNA-seq).

For inducing cell differentiating toward the endoderm, the cells were cultured in RPMI/1640 medium supplemented with 100 ng/ml Activin A (PeproTech, 120–14E), 50 ng/ml WNT3A (R&D systems, 5036-WN), 0.5% FBS, 200 mM GlutaMAX, 0.2× MEM non-essential amino acids solution and 55 μM 2-mercaptoethanol for a duration of 5 days. At day 5, the cells were collected for real-time RT-PCR analysis and total RNA-seq.

For inducing cell differentiating toward the ectoderm, the cells were cultured in DMEM/F12 medium supplemented with 2 μM TGF-b inhibitor A83-01 (Tocris, 2939), 2 μM WNT3A inhibitor PNU-74654 (Tocris, 3534), 2 μM dorsomorphin BMP inhibitor (Tocris, 3093), 15% Knockout™ SR (Gibco, 10828028), 0.2× MEM non-essential amino acids solution and 55 μM 2-mercaptoethanol for a duration of 5 days. At day 5, the cells were collected for real-time RT-PCR analysis and total RNA-seq.

### Human ESCs-to-mesoderm differentiation

The differentiation of early mesoderm lineage was performed following the methodology described in our previous study (28). In brief, hPSCs were dissociated into single cells using Accutase (Sigma, A6964), and then plated at a density of 2.5 × 10^5^ cells on Matrigel-coated 12-well cell culture plates in mTeSR1 medium with 10 μM Y27632 for 24 h, and then changed to mTeSR1 medium without Y27632 for the subsequent 2 days until the cell density up to 90% confluency. At day 0, these cells were treated with RPMI/1640 medium containing 6 μM Laduviglusib (CHIR99021) (Selleckchem, S1263) for 24 h. At day 1, the medium was replaced with only pure RPMI/1640 medium for 48 h. At day 3, the cell was identified as being of early mesoderm lineage.

### Immunofluorescence

The cells growned on coverslips were washed 3 times with phosphate buffered saline (PBS), then fixed with 4% paraformaldehyde (PFA) for 30 min at room temperature. Subsequently, they were permeabilized with 0.1% Triton X-100 in PBS, and blocked with 3% bovine serum albumin (BSA) for 30 min. The coverslip was then incubated with a primary antibody at 4°C overnight. The primary antibodies in this assay were as follows: Rabbit anti-S100 Alpha 6/PRA (Abcam, ab181975, 1:400), Rabbit anti-OCT4 (Abcam, ab19857, 1:1000), Rabbit anti-SOX2 (Abcam, ab97959, 1:1000), Rabbit anti-c-JUN (CST, 9165, 1:400), Rabbit anti-EOMES (Affinity, DF8543, 1:400) and Rabbit anti-GATA4 (Abcam, ab307823, 1:50). After incubating, the coverslip was washed 3 times with PBS, then incubated with a secondary antibody Goat anti-Rabbit Alexa Flour 488 (Thermo Fisher Scientific, A-11008, 1:500) for 1 h at room temperature, washed 3 times with PBS, incubated with DAPI (Beyotime, C1002, 1:5000) for 5 min and washed twice with PBS. Finally, after the coverslips were gently dried, they were mounted on slides. The cells were observed and imaged using a laser confocal microscope ZEISS LSM 800 or Olympus FV3000.

### Flow cytometry

The digested cell suspension was filtered through a 70 μm cell filter (BD Biosciences) to obtain a single cell suspension. The suspension was fixed with 4% PFA for 20 min, followed by wash twice with PBS (at least 5 min each time), then blocked with 1% BSA, permeabilized with 0.2% Triton X-100 for 10 min and washed 3 times in PBS. The cells were then incubated with antibody at 4°C overnight, then washed twice with PBS. The antibodies were used as follows: Rabbit anti-S100 Alpha 6/PRA (Abcam, ab181975, 1:400), Rabbit anti-PDGFRA (Affinity, AF0241, 1:500), Mouse anti-HAND1 Alexa Fluor® 647 (Novus, NBP2-71459AF647, 1:400) and Mouse anti-CD326 (EpCAM) Alexa Fluor® 488 (BioLegend, 324210, 1:400). The secondary antibody if necessary, was Goat anti-Rabbit Alexa Flour 488 (Thermo Fisher Scientific, A-11008, 1:500) for 1 h at room temperature. After incubating, the cells were washed twice with PBS. Finally, the cells were analyzed with the Accuri® C6 Plus flow cytometer (BD Biosciences), and the resulting data were processed using FlowJo software.

### Western blotting

Total proteins from the cultured cells were lysed in RIPA buffer (Beyotime) containing an ethylenediaminetetraacetic acid-free proteinase inhibitor cocktail (Roche, 4693132001) on ice for 30 min. The supernatant samples were collected by centrifuging for 15 min at 4°C, 12 000 r.p.m. Protein concentration was measured using the BCA protein assay kit (Thermo Fisher Scientific, 23227). Proteins were separated by 10–12% sodium dodecyl sulfate–polyacrylamide gel electrophoresis, then transferred to the Polyvinylidene Fluoride (PVDF) membrane, and blocked with 5% fat-free milk in Tris Buffered Saline with Tween-20 (TBST) for 2 h at room temperature. Primary antibodies were incubated at 4°C overnight, then washed 3 times with TBST and then incubated with their corresponding horseradish peroxidase (HRP)-conjugated secondary antibody for 1 h at room temperature. The membranes were washed twice with TBST and once with Tris Buffered Saline (TBS). The primary antibodies used in this study were as follows: Rabbit anti-S100 Alpha 6/PRA (Abcam, ab181975, 1:1000), Rabbit anti-c-JUN (CST, 9165, 1:1000), Rabbit anti-EOMES (Affinity, DF8543, 1:1000), Rabbit anti-GATA4 (Abcam, ab307823, 1:1000), Rabbit anti-β-Catenin (Abcam, ab32572, 1:2000) and Rabbit anti-MBD3 (Abcam, ab157464, 1:1000). Rabbit anti-GAPDH HRP Conjugate (CST, 8884, 1:2000), Rabbit anti-Histone H3 (Abcam, ab1791, 1:2000) and Rabbit anti-β-Actin HRP Conjugate (CST, 5125, 1:1000) were employed as a loading control. The secondary antibody used in this study was as follows: Goat anti-Rabbit IgG (H+L) Secondary Antibody HRP (Thermo Fisher Scientific, 31460, 1:10 000). Finally, the protein bands were visualized using chemiluminescence (ECL, Merck Millipore, WBULS0100).

### Co-immunoprecipitation

The HEK293T cells were transfected using Lipofectamine 3000 (Thermo Fisher Scientific, L3000001) following the manufacturer’s guidelines, when they reached ∼80% density. Plasmids expressing PB-CAG-*c-JUN*-Flag and PB-CAG-*MBD3*-HA, PB-CAG-*c-JUN*-Flag and PB-CAG-*MTA2*-HA and PB-CAG-*c-JUN*-Flag and PB-CAG-*MTA*3-HA were, respectively, co-transfected into the HEK293T cells. At 36 h post-transfection, the cells were collected and lysed with RIPA lysis buffer (Beyotime) containing a protease inhibitor cocktail (Roche, 4693132001) for 30 min on ice, then centrifuged at 12 000 r.p.m. for 15 min at 4°C. The supernatant was transferred to a new tube. Then, relative primary antibody or control IgG: anti-Rabbit IgG antibody (Abcam, ab6702, 1:3000) and anti-Mouse IgG antibody (Abcam, ab6708, 1:3000), were incubated with the supernatant, rotating overnight at 4°C. The primary antibody for immunoprecipitation was performed using Mouse anti-Flag (Sigma, F1804, 1:100) and Rabbit anti-HA antibody (CST, 3724s, 1:50). The next day, protein A beads (Thermo Fisher Scientific, 10008D) and protein G beads (Thermo Fisher Scientific, 10009D) were added to the solution, shaking at 4°C for 2 h. After washing 3 times, magnetic beads were suspended with the elution buffer, and boiled at 100°C for 10 min, the suspended samples were subjected to western blot analysis. The secondary antibodies used in this assay were as follows: Goat anti-Rabbit IgG (H+L) Secondary Antibody HRP (Thermo Fisher Scientific, 31460, 1:10 000) and Goat anti-Mouse IgG (H+L) Secondary antibody HRP (Thermo Fisher Scientific, 31430, 1:10 000).

### Production of lentiviruses and construction of human MBD3 knockdown cell line

HEK293T cells were seeded at an approximately density of 4 × 10^6^ cells per 10 cm dish for packaging lentivirus. The cells were transfected using Polyethylenimine Transfection Reagent (PEI) transfection reagent (Polysciences, 23966). Briefly, lentiviral transfer plasmids (6 μg pLKO.1-U6-shRNA *MBD3*-Puro), a packaging plasmid pMD2.G (1.5 μg, Addgene, #12260) and psPAX2 (4.5 μg, Addgene, #12259) were mixed with 36 μg PEI transfection reagent in Opti-MEM medium. The pLKO.1-scramble shRNA plasmid served as the negative control vector. Subsequently, the mixture was incubated for 30 min at room temperature and added drop-by-drop to the HEK293T cells in 10 ml fresh culture medium. Supernatants containing lentiviruses were, respectively, harvested at 48 and 72 h after transfection, and filtered using a 0.45-μm filter. Then the virus was concentrated using the Lenti-X Concentrator (TaKaRa, 631231) reagent according to the manufacturer’s instruction. The virus pellets were resuspended in mTesR1 medium and stored at −80°C for long-term storage. The viruses were purified in a BSL-2 laboratory following safety protocols for handling biological materials. The shRNA sequence of human *MBD3* was listed in supplementary table.

The hPSCs (H1) cell lines were employed to establish the *MBD3* knockdown cell line. The cells were plated at appropriate density into six-well plates in mTesR1 with 10 μM Y-27632. On the next day (day 1), the suitable amount of purified virus was added into culture medium mTesR1 without Y-27632 supplemented with 5 μg/ml polybrene (Biosharp, BL628A). After 24 h of virus infection (day 2), the medium was changed to fresh mTesR1 medium. On day 3, puromycin was used to select the virus infected cells. After three passages following with puromycin selecting, the cells were harvested to assess the MBD3 gene expression level by Reverse Transcription Quantitative Polymerase Chain Reaction (RT-qPCR) to identify the efficiency of knockdown. And the knockdown cell line was used for the further study.

### Real-time RT-PCR and total RNA-seq

The total RNA was extracted using the RNA isolater Total RNA Extraction Reagent (Vazyme, R401-01), following the provided instructions. Subsequently, complementaryDNA (cDNA) was synthesized from the RNA using HiScript III RT SuperMix (+gDNA wiper) (Vazyme, R323-01). The real-time PCR analysis was conducted using ChamQ SYBR qPCR Master Mix (Vazyme, Q311-02) on a QuantStudio 3 system (Thermo Fisher Scientific). Each sample was analyzed in triplicate, with GAPDH used as the internal reference, and the relative expression was calculated according to the comparative cycle threshold (ΔΔCt) method. The specific primers used for real-time RT-PCR were provided in supplementary table.

For RNA-seq, the total RNA was acquired as described above. Each sample required 1 μg RNA for constructing the RNA library. The construction of the total RNA library used the VAHTSTM Total RNA-seq (H/M/R) Library Prep Kit for Illumina® (Vazyme, NR604). Initially, ribosomal RNA was removed using the Ribo-off Depletion Kit, followed by messenger RNA (mRNA) capture beads to purify the ribosomal-depleted RNA. The RNA was then fragmented using Frag/Prime Buffer at 94°C for 8 min. Subsequently, double stranded cDNA was synthesized, and adapters were ligated. DNA clean beads were used to purify the fragment, and PCR amplification was carried out for 15 cycles to obtain the RNA library. All libraries were sequenced on an Illumina NovaSeq 6000 system.

RNA-seq data were processed as described in previous studies (30,31). Briefly, reads were aligned to a transcriptome generated using the hg38 genome and GENCODE annotations using Bowtie2 (32). The resulting read/gene matrices were standardized through the use of RSEM (33) and EDASeq (34,35). RNA-seq data were expressed in GC normalized label count units. Differentially expressed genes (DEGs) were analyzed by DESeq2 (35).

### ATAC-seq

ATAC sequencing (ATAC-seq) was carried out following the methods outlined previously (36). Briefly, 5 × 10^4^ cells were washed with 50 μl of cold PBS and then centrifuged at 500 × *g* for 5 min at 4°C. Then the cell pellet was suspended in 50 μl of cold lysis buffer [10 mM Tris–HCl pH 7.4, 10 mM NaCl, 3 mM MgCl_2_, 0.1% (v/v) IGEPAL CA-630 (Sigma, I8896)] on ice for 10 min and centrifuged at 500 × *g* for 5 min at 4°C. Following, the sample was added to 50 μl transposition reaction mix (25 μl Tagment DNA (TD) buffer, 2.5 μl Tn5 transposase and 22.5 μl nuclease-free H_2_O) from the Nextera DNA library Preparation Kit (Illumina, FC-121–1031), and incubated at 37°C for 30 min. DNA was extracted using a MinElute Kit (QIAGEN, 28006), and eluted in 10 μl elution buffer. For library amplification, 10 μl of purified DNA was mixed with 25 μl NEBNext High-Fidelity 2× PCR Master Mix buffer along with 2.5 μl barcoded i5 and i7 primers. A total volume of 50 μl of sample was amplificated with the following program: 72°C for 5 min; 98°C for 30 s; 12 cycles of 98°C for 10 s, 63°C for 30 s; 72°C for 1 min; and holding at 4°C. To purify the PCR products, DNA was isolated using a MinElute Kit (QIAGEN, 28006), and eluted in 20 μl elution buffer. All ATAC-seq libraries were sequenced using the Illumina NovaSeq 6000 platform.

### CUT&RUN

The CUT&RUN (cleavage under targets and release using nuclease) assay was performed using Hyperactive pG-MNase CUT&RUN Assay Kit for Illumina (Vazyme, HD102) according to the provided guidelines. Briefly, 1 × 10^5^ cells were collected, washed once with 500 μl wash buffer and centrifuged at 600 × *g* for 5 min. The cell pellets were resuspended in 100 μl wash buffer and incubated with ConA beads for 10 min with rotation at room temperature. Following this, cells were incubated with an antibody at 4°C overnight with gentle rotation. Then cells were washed 3 times with Dig-wash buffer and incubated with pG-MNase for 1 h, at 4°C. Subsequently, cells were washed 3 times with Dig-wash buffer, resuspended in tagmentation buffer and incubated at 37°C for 1.5 h. Tagmentation was stopped by adding stop buffer and incubating at 37°C for 30 min. Then DNA was extracted, and libraries were constructed using adapter VAHTS Multiplex Oligos Set 4/5 for Illumina (Vazyme, N321). All libraries were sequenced on an Illumina NovaSeq 6000 system.

### Single-cell RNA-seq and data analysis

The cells were collected and resuspended in DPBS solution with 0.04% BSA. Cell suspensions containing 500–1000 cells per microliter were loaded onto a chromium single cell controller (10x genomics). Single cell gel beads in emulsion (GEMs) were obtained by using Single Cell 3′ Library and Gel Bead Kit V2 (10x genomics, 120237). The captured cDNA was lysed and the released RNAs were barcoded through reverse transcription in singular GEMs. Barcoded cDNAs were pooled and cleaned by DynaBeads® MyOne Silane Beads (Invitrogen, 37002D). Single-cell RNA-seq (scRNA-seq) libraries were prepared by Single Cell 3′ Library Gel Bead Kit V2 (10x genomics, 120237) following the manufacturer’s instruction. Sequencing was performed on an Illumina HiSeq X Ten with pair end 150-bp reads (PE150).

Fastq reads were aligned to the genome using STAR (37,38) with the setting ‘ –soloType Droplet –soloFeatures Gene Velocyto –runThreadN 20 –soloCBstart 1 –soloCBlen 16 –soloUMIstart 17 –soloUMIlen 12 –soloBarcodeReadLength 0 –readFilesCommand zcat –outSAMtype BAM SortedByCoordinate –outSAMattributes NH HI AS nM CR CY UR UY’. The count matrix was filtered to exclude cell barcodes with low numbers of counts: cells with <2000 UMIs, <500 genes or >20% fraction of mitochondrial counts were removed. The filtered matrix was normalized according scanpy tutorials. The top 3000 most highly variable genes were used for principal component analysis (PCA), and the first six principal components were used for downstream analysis with scanpy. The predicted differentiation score was calculated by CytoTRACE (39). RNA velocity measurements were generated with scVelo (40,41). Cell ordering and pseudotime were calculated by Monocle2 (42).

### ATAC-seq bioinformatic analysis and peak calling

All sequencing data were mapped to the hg38 human genome assembly using Bowtie2 with the options –very-sensitive. samtools was used to remove low-quality mapped reads with option view –q 35, and unique reads were kept. We removed mitochondrial sequences using “grep –v ‘chrM’”. Biological replicates were merged, and peaks were called using dfilter (43) (with the settings: –bs = 100 –ks = 60 –refine –std = 5). BigWig files were produced using genomeCoverageBed from bedtools (scale = 107/<one sample’s total unique reads>) and then bedGraphToBigWig. Gene ontology (GO) and gene expression measures were called by first collecting all transcription start sites within 10 kb of an ATAC-seq peak and then performing GO analysis with goseq (44), or measuring gene expression. Other analysis was performed using GLBase (30).

### Recalling weak peaks from the ATAC-seq data

Peak recalling is based on the method we previously described (36). Briefly, when dfilter is used to discover peaks, as described above, it is generally conservative and will not call a weak peak. Hence, we ‘re-call’ all peaks by measuring the sequence tag density in all ATAC-seq libraries for all possible peaks in any other ATAC-seq library, irrespective of which library the peak was called in by dfilter (43). Then we get a superset of all possible peaks in any library, and based on our previous analysis, we used an arbitrary minimum threshold of 0.2734 to filter out false peaks; if the ATAC-seq is below this value, it is annotated as ‘closed’ and above ‘open’. All downstream analysis was based on this new peak list.

### ChIP-seq data analysis

Reads from Chromatin Immunoprecipitation Sequencing (ChIP-seq) experiments were mapped to the mouse genome (hg38) using Bowtie2 (–very-sensitive), as described for ATAC-seq data, and only the uniquely mapped reads were kept for further analysis. Peaks were called using MACS2 (45) software with the default parameters.

### Computational analysis of TF binding sites and open chromatin regions

Motif analysis was performed by HOMER 28 with default settings. Motifs were only kept if the *P*-value was <0.01 and (<percent of target>/<percent of background>) was >1.5. Annotation of the ChIP-seq/ATAC-seq peaks/open chromatin to genes was performed by HOMER using default settings.

### Mass spectrometry and analysis

The mass spectrometry (MS) experiment utilized Acclaim PepMap 100 C18 columns for data collection and the time consumed in collecting total data was 140 min. Gradient B buffer (aqueous solution of 80% acetonitrile and 0.1% formic acid) was used for peptide separation (2–22% for 100 min, 22–28% for 20 min and 28–36% for 12 min). Subsequently, the B buffer was used to wash twice more (2 min of 36–100% and 6 min of 100%). The Easy nLC 1200 was connected online to a Fusion Lumos mass spectrometer (Thermo Fisher Scientific). Scans were collected in data-dependent maximum speed mode with a dynamic exclusion time of 90 s. MaxQuant version 1.6.0.1 was used to search a human protein FASTA database for analyzing raw data, with label free quantification and match between runs functions enabled. The DEP package was utilized to analyze and visualize the output proteome (46).

## Results

### Human *c-JUN* restricts mesoderm and endoderm lineages *in vitro*

**Figure 1.**
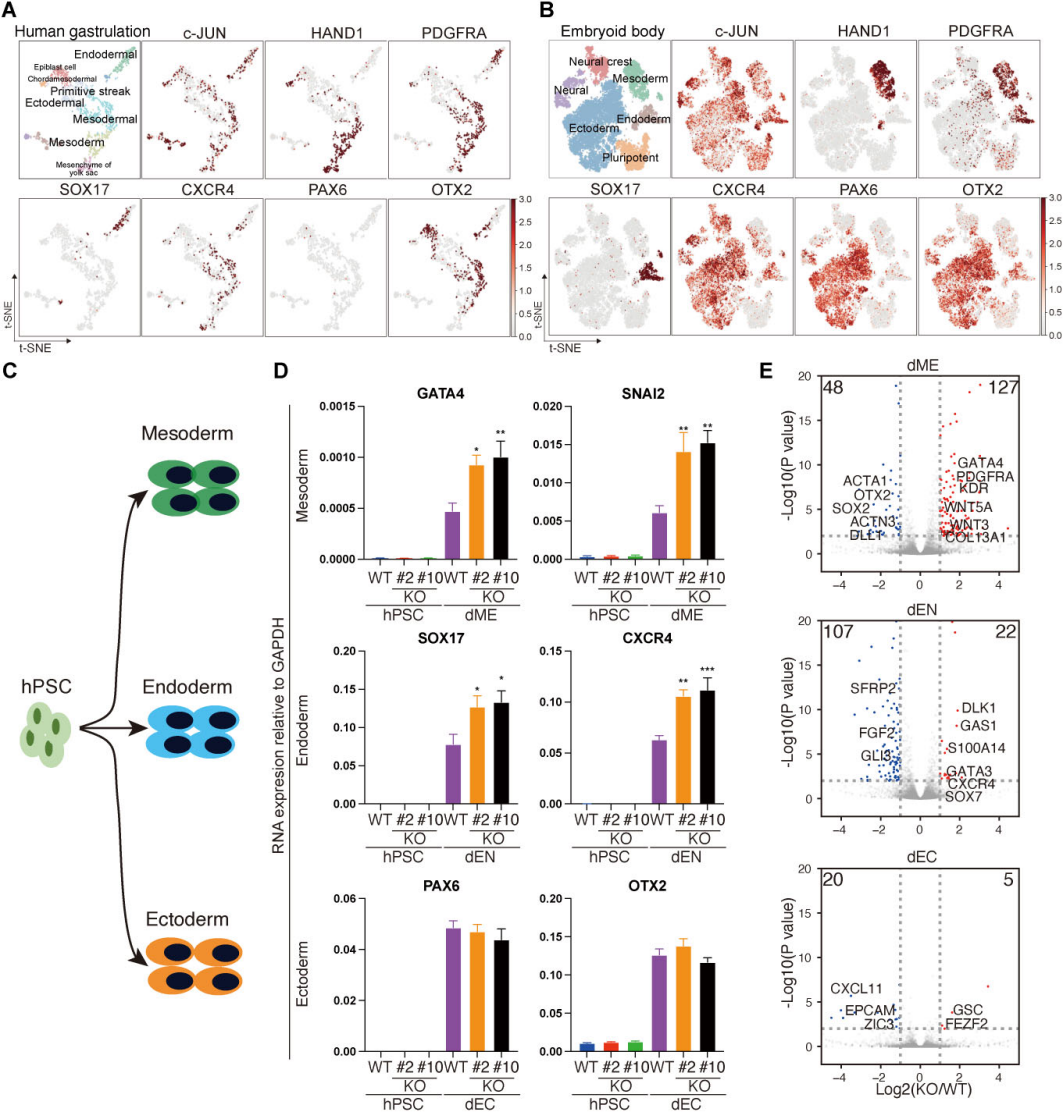
*c-JUN* regulates germ layer differentiation. (**A**) t-SNE plots for germ layer marker genes from human gastrulating embryo scRNA-seq data. Data and cell type designations were taken from the original publication (48). (**B**) t-SNE plots of scRNA-seq data from *in vitro* differentiated hPSC separately differentiated to the indicated cell types using defined culture conditions. Data are from published data (49), and cell type designations were taken from that study. (**C**) Schematic showing the directed differentiation of hPSC to early germ lineage cells. (**D**) Reverse Transcription Quantitative Polymerase Chain Reaction (RT-qPCR) for selected maker genes for the indicated lineages in hPSCs (day 0) versus day 5 of differentiation in WT or *c-JUN* KO cells. Data were from six to nine biological replicates in three independent experiments and are shown as the mean ± SEM (standard error of the mean). ***P-value < 0.001, **P-value < 0.01 and *P-value < 0.05 from a one-way ANOVA with Sidak correction between the WT hPSCs and *c-JUN* KO groups. (**E**) Volcano plot shows the DEGs between WT and *c-JUN* KO cells on day 5 of the indicated differentiation treatment. Genes were considered significantly different if their q-value was <0.05 and their absolute fold change (FC) was >2.0.

Previously, we have shown that *c-JUN* acts as a barrier during the reprogramming of MEFs into iPSCs and the development of cardiomyocytes (23,26,28,47). To understand how *c-JUN* regulates somatic cell fate, especially the establishment of the germ layer lineages upon exit from pluripotency, we reanalyzed published scRNA-seq datasets from *in vitro* differentiation systems and human gastrulation (48,49). We found *c-JUN* was widely expressed during early embryonic development, as evidenced by both *in vitro* and *in vivo* expression data (Figure 1A and B, and Supplementary Figure S1A and B). However, the necessity of *c-JUN* during lineage commitment in early human embryonic development and its role in germline cell fate regulation require further investigation. To unravel the role of *c-JUN* in early human embryonic development, we generated two independent c-*JUN* knockout hPSC (H1) lines (*c-JUN*^−/−^ H1 #2 and #10) without disrupting their normal karyotype or pluripotency markers (Supplementary Figure S1C–F). Differentiating these hPSCs into the three primary germ layers—mesoderm, endoderm and ectoderm—using established protocols (3), we observed that the knockout of *c-JUN* led to the activation of mesoderm markers such as *GATA4* and *SNAI2* (Figure 1C and D). Consistent with a previous study (27), c-*JUN* KO also upregulated endoderm markers like *SOX17* and *CXCR4*, while it had no significant effect on ectoderm markers *PAX6* and *OTX2* (Figure 1D). A comparative analysis of DEGs between c-*JUN* KO and wild-type (WT) cells and their differentiated progeny: differentiated mesoderm (dME), differentiated endoderm (dEN) and differentiated ectoderm (dEC), we found that *c-JUN* KO activated 127 genes in dME, including key factors such as *GATA4*, *PDGFRA* and *KDR*, and enhanced 22 genes upregulated in dEN, including the endoderm-specific marker *SOX17* and *CXCR4 (*Figure 1E). This suggests that KO *c-JUN* enhances dME and dEN generation (27). In contrast, the effect on dEC was minimal, with <20 genes differentially affected (Figure 1E). Taken together, *c-JUN* acts as a regulatory barrier for mesoderm and endoderm lineages, but not for the early ectoderm lineage.

### *c-JUN* is a barrier during mesoderm generation

**Figure 2.**
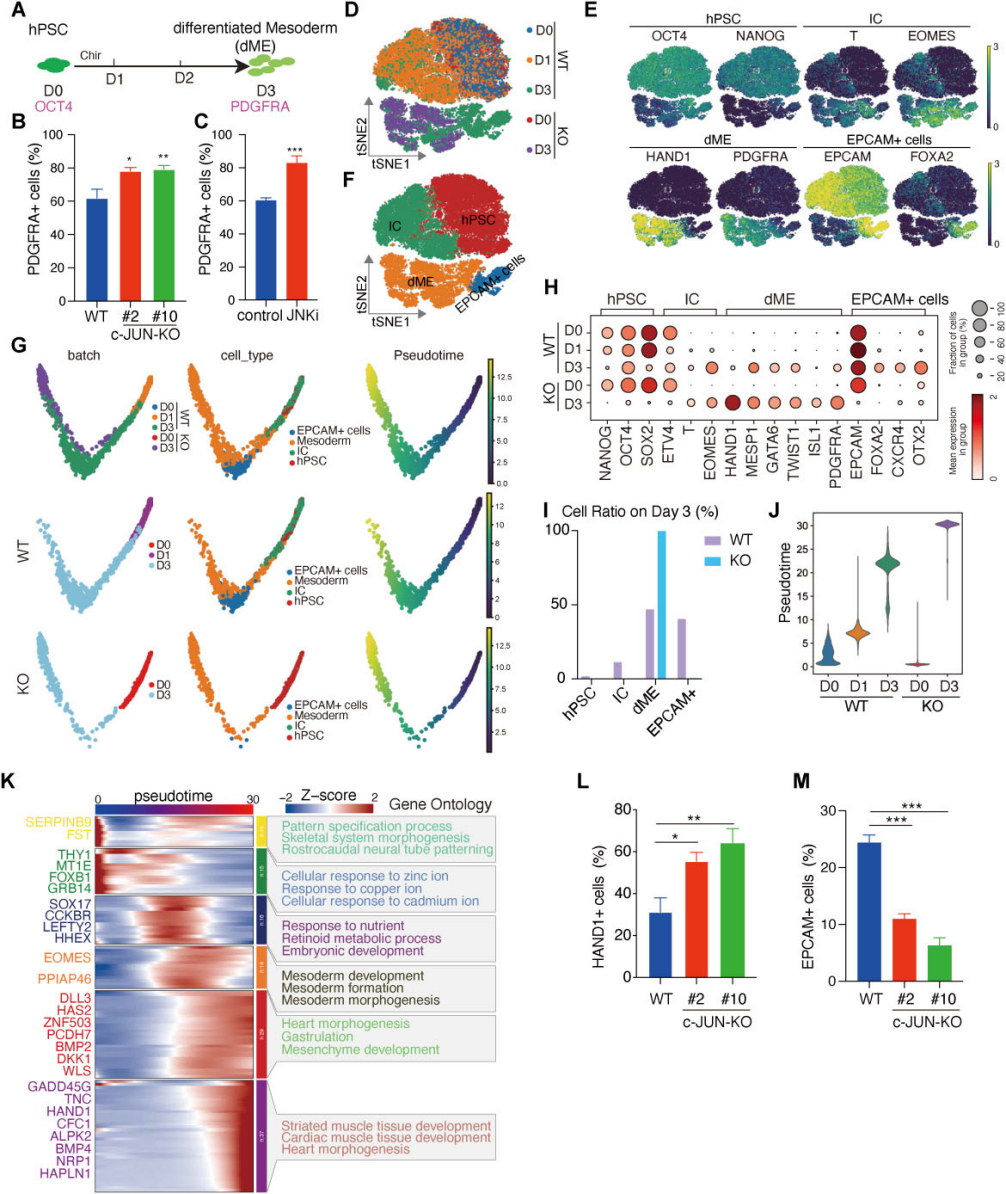
*c-JUN* inhibits mesoderm development. (**A**) Schematic of induction hPSC differentiation into mesoderm cells. D = Day. (**B**) Flow cytometry quantification of mesoderm cells’ differentiation efficiency. Knockout of *c-JUN* enhances the generation of *PDGFRA*+ cells after 3 days of differentiation. Data were from six biological replicates in two independent experiments and are shown as the mean ± SEM. **P-value < 0.01, *P-value < 0.05, one-way ANOVA with Sidak correction between the WT and *c-JUN* KO groups. (**C**) Flow cytometry quantification of mesoderm cells’ differentiation efficiency. Small molecular JNKi (SP600125, 2 μm) enhances the generation of *PDGFRA*+ cells during 3 days of induction. Data were from seven biological replicates in three independent experiments and are shown as the mean ± SEM. ***P-value < 0.001, unpaired *t*-test of control (Dimethyl Sulfoxide, DMSO) and JNKi groups. DMSO and JNKi were added into the cell culture medium and changed every day. (**D**) t-SNE showing the scRNA-seq data of WT and *c-JUN* KO cells during hPSC differentiation into mesoderm. (**E**) t-SNE plots showing the expression level of representative marker genes during the induction of hPSC into mesoderm. (**F**) Four cell clusters were defined according to the specific marker genes. (**G**) Cell ordering on hPSC-to-mesoderm transition arranged them into a major trajectory, annotated cell type with the ordered trajectory, pseudotime ordering on hPSC-to-mesoderm transition. (**H**) Dotplot shows the expression of the marker genes of four cell types in WT and *c-JUN* KO conditions. (**I**) Percentage of each cell type in each day between WT and *c-JUN* KO condition. (**J**) Violin plot shows the different differentiated states of WT and *c-JUN* KO cells. (**K**) A heatmap with all dynamically expressed genes ordered along pseudotime that illustrates the waves of gene expression underlying differentiation. Significantly enriched GO terms for clusters are labeled. (**L**) Flow cytometry quantification of mesoderm differentiation efficiency. Knockout of *c-JUN* enhances the generation of *HAND1*+ cells during 3 days of induction. Data were from six biological replicates in two independent experiments and are shown as the mean ± SEM. **P-value < 0.01, *P-value < 0.05, one-way ANOVA with Sidak correction between the WT control and *c-JUN* KO groups. (**M**) Flow cytometry quantification of *EPCAM*+ cells during iME formation. Knockout of *c-JUN* reduces the generation of *EPCAM*+ cells during 3 days of induction. Data were from six biological replicates in two independent experiments and are shown as the mean ± SEM. ***P-value < 0.001, one-way ANOVA with Sidak correction between the WT control and *c-JUN* KO groups.

To further investigate the role of *c-JUN* in cell fate regulation, we focused on mesoderm differentiation. We adopted a 3-day mesoderm induction system (50), which includes a 1-day CHIR99021 treatment to activate the WNT signaling pathway. This system promotes the differentiation of hPSCs into a population of *PDGFRA*+ mesoderm cells (55.7%) (Figure 2A, and Supplementary Figure S2A). Although this method showed lower mesoderm differentiation efficiency compared with the 5-days differentiation protocol (3) [∼70% as reported in our recently publication (51)], it resulted in a faster cell fate transition, making the influence of *c-JUN* more readily observable during the transition of hPSCs into mesoderm. Transcriptome data reflected the establishment of the mesoderm gene expression program, as evidenced by the downregulation of pluripotency genes *NANOG* and *UTF1*, and the upregulation of mesoderm genes *EOMES*, *GATA4* and *PDGFRA* (Supplementary Figure S2B and C). We induced mesoderm differentiation in both c-*JUN* KO and WT hPSCs and quantified the proportion of *PDGFRA*+ cells (Figure 2B). As expected, the proportion of *PDGFRA*+ cells were increased in *c-JUN* KO cells, and this phenotype could be replicated with a JNK inhibitor (JNKi; SP600125) (Figure 2C). Interestingly, our findings show that *c-JUN* is initially downregulated on day 1 (D1) but then is reactivated by day 3 (Supplementary Figure S2D–F), suggesting that *c-JUN* needed be repressed during the early stage of hPSCs differentiation into mesoderm (29).

PCA of transcriptome changes between WT and *c-JUN* KO cells revealed a distinct trajectory that emerges following *c-JUN* knockout (Supplementary Figure S3A and B). Analysis of DEGs between WT and *c-JUN* KO cells from D0 to D3 showed that knocking out *c-JUN* did not affect the expression profile of hPSCs (D0), but its absence triggered the activation of *T* and *MESP1*, two genes associated with early mesoderm differentiation (Supplementary Figure S3B). This effect extended to the elevated expression of mesoderm-related genes such as *HAND1*, *PDGFRA*, *GATA4* and *TBX3* on D3 (Supplementary Figure S3B and C). In summary, these results suggest that *c-JUN* plays a crucial role in restricting mesoderm formation.

To investigate the inhibitory role of *c-JUN* on mesoderm commitment, we performed scRNA-seq of hPSCs (D0), and day 1 and day 3 cells (28) (Figure 2D). Using t-distributed stochastic neighbor embedding (t-SNE) analysis, we clustered the cells into four distinct cell types: hPSCs, intermediate cells, *EPCAM*+ cells and dME (Figure 2E–G, and Supplementary Figure S3D). We observed a pronounced downregulation of pluripotency genes, including *NANOG* and *POU5F1* (*OCT4*), and an upregulation of mesoderm genes, such as *HAND1* and *ISL1* at D3 in *c-JUN* KO cells (Figure 2H). Based on the defined cell clusters, we analyzed the cell population at D3 and found that the dME percentage stood at 99.5% in *c-JUN* KO cells compared with 46.8% WT cells (Figure 2I), suggesting that *c-JUN* KO resulted in a more homogenous mesoderm population.

To further dissect the kinetics of hPSCs differentiation into mesoderm, we employed the Monocle2 algorithm (17) to compute cell trajectories (Figure 2G). This analysis suggested a trajectory from hPSCs to mesoderm. Pseudotime analysis indicated that *c-JUN* knockout cells not only prompted hPSCs to differentiate into mature mesoderm but also prematurely activated the gene regulatory network associated with cardiac precursor cells (Figure 2J and K). This observation was consistent with flow cytometry of *PDGFRA*+ cells (Figure 2B) and was further validated by flow cytometry assays targeting another mesoderm marker *HAND1* (Figure 2L, and Supplementary Figure S3E). Under WT conditions at D3, a population of *EPCAM*+ cells emerged (Figure 2E and I), expressing *EPCAM* and *OTX2* (Figure 2H). However, *c-JUN* KO repressed the *EPCAM*+ cell population due to the loss of *c-JUN* binding and subsequent downregulation of *EPCAM* expression (Figure 2I and M, and Supplementary Figure S3F and G). In summary, these findings indicate that *c-JUN* hinders the differentiation of dME.

### c-JUN recruits MBD3 to silence mesoderm genes

**Figure 3.**
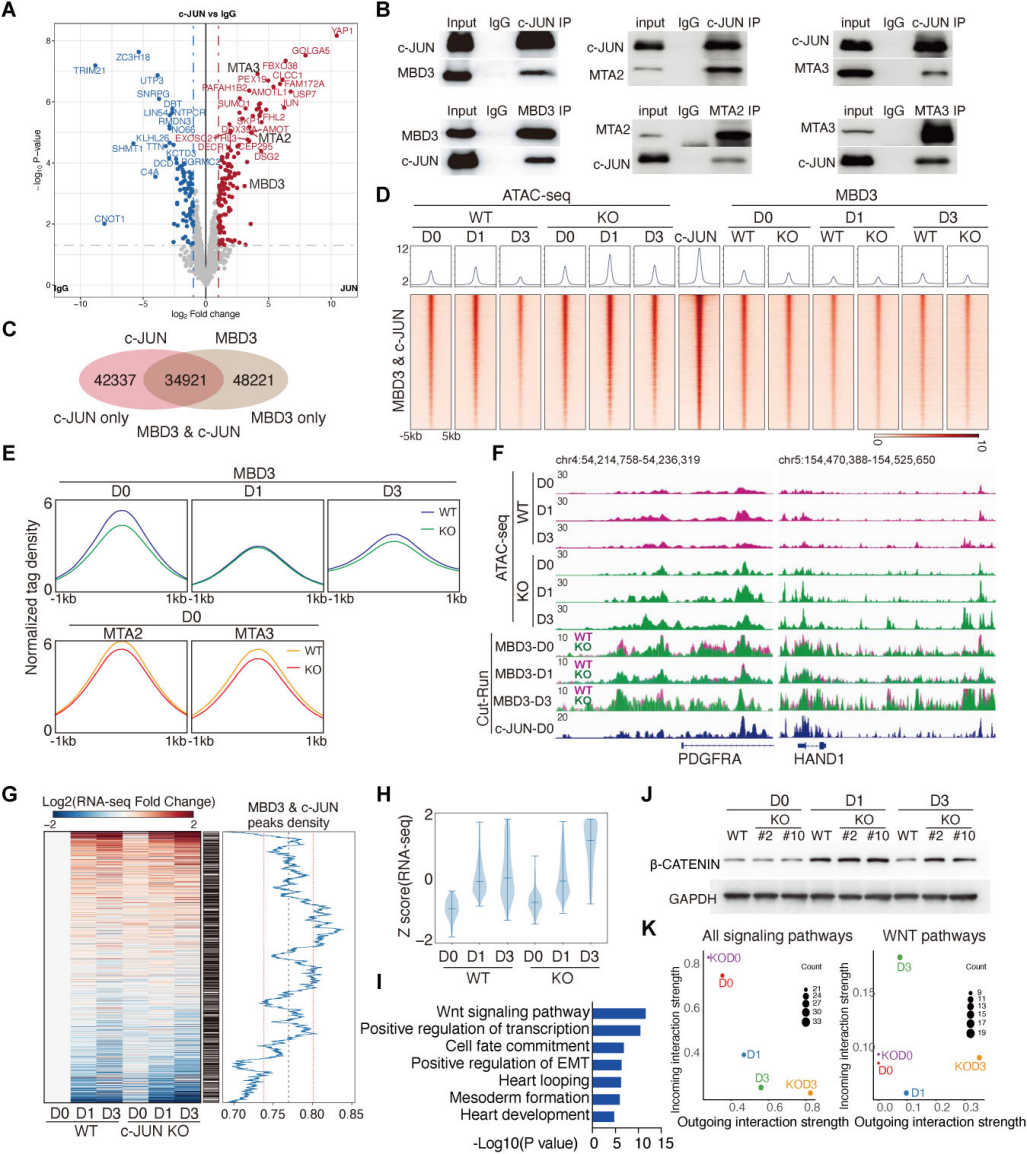
c-JUN recruits MBD3 to maintain chromatin accessibility. (**A**) Volcano plot for c-JUN interacting proteins in hPSC. Target proteins were shown in the plot with FC ≥ 2 and *P*-value < 0.05. (**B**) Co-IP of Flag-tagged c-JUN with MBD3, MTA2 and MTA3 in 293T cells. IgG: Immunoglobulin G. (**C**) Venn plot shows the overlap binding loci of c-JUN and MBD3 in hPSC. (**D**) Heatmap shows the signal of WT and *c-JUN* KO ATAC-seq data and c-JUN, MBD3 CUT&RUN data in hPSC and D1 induction cells with or without *c-JUN* on MBD3 and c-JUN co-binding loci defined in panel (C). (**E**) Average CUT&RUN sequence read density of MBD3, MTA2 and MTA3 data in hPSC (D0) and D1, D3 cells during induction hPSC differentiation into mesoderm, the pileups are centered on MBD3 & c-JUN co-binding loci. (**F**) Genome view of the ATAC-seq data and MBD3, c-JUN CUT&RUN data of hPSC and D1, D3 cells on select target genes. (**G**) The co-binding loci of c-JUN and MBD3 are correlated with upregulated genes. Gene expression was ranked from up to downregulated (left), and the FC value of each RNA-seq data was related to WT D0. c-JUN and MBD3 co-binding was marked as present if found within 5 kb (middle); the density of c-JUN and MBD3 co-binding was then averaged using a moving-average window to visualize the binding density of c-JUN and MBD3 across the gene changes (right). (**H**) Violin plot shows that c-JUN and MBD3 co-binding genes were highly expressed in D3 when *c-JUN* was knocked out. (**I**) GO analysis shows the genes in panel (H) were related to mesoderm formation and heart development. (**J**) Western blot shows β-CATENIN protein level during hPSC-to-mesoderm transition in WT and *c-JUN* KO cells. (**K**) Scatter plot shows the incoming and outcoming interaction strength in both signaling pathways and WNT pathways. The data were calculated with scRNA-seq data by CellChat (55).

Previous studies have shown that *c-JUN* influences chromatin accessibility and thereby dictates cell fate (26,27,52). To identify c-JUN’s interacting partners, we performed co-immunoprecipitation (Co-IP) followed by MS. Intriguingly, c-JUN interacted with components of the NuRD complex, specifically MTA2/3 and MBD3 (Figure 3A). This interaction was confirmed by Co-IP western blot (Figure 3B). The NuRD complex plays a crucial role in epigenetic regulation during cell fate decisions, orchestrating chromatin modifications that regulate gene expression (53,54). We posited that c-JUN might guide MBD3 binding to specific target genes. To test this hypothesis, we conducted CUT&RUN assays for MBD3 in hPSCs. Remarkably, 45% of the c-JUN binding sites (34 921 loci) overlapped with MBD3 binding sites (Figure 3C). Knockout of *c-JUN* led to enhanced opening of c-JUN & MBD3 co-bound loci from D0 to D3 (Figure 3D). Conversely, *c-JUN* knockout reduced MBD3 binding in hPSCs and D3, and weakening observed at D1, aligning with the reduced c-JUN protein level in the WT cells as well (Figure 3D and E, and Supplementary Figure S2E). *c-JUN* KO also affects *MBD3* targeted genes during hPSCs-to-mesoderm transition (Supplementary Figure S4A). Collectively, these findings underscore the interaction between c-JUN and the MBD3–NuRD complex in orchestrating chromatin accessibility during the exit from pluripotency.

Chromatin remodeling precedes the establishment of gene expression programs (26). The mesoderm marker genes *PDGFRA* and *HAND1*, which are bound by c-JUN, exhibited increased chromatin accessibility, coupled with slight reduced MBD3 binding in response to *c-JUN* deficiency (Figure 3F). We analyzed the expression profiles of genes located within 5 kb upstream and downstream of c-JUN & MBD3 co-binding sites, and genes bound by MBD3 and c-JUN tended to be associated with upregulated genes (Figure 3G). Among those target genes, 534 genes exhibited slight activation in *c-JUN* knockout in hPSC (D0), followed by increased expression levels at D1 and D3 (Figure 3H). GO analysis indicated that these genes are involved in crucial processes such as the WNT signaling pathway, cell fate commitment and mesoderm formation (Figure 3H and I). The elevated activation of these pathways indicates robust mesodermal development in *c-JUN* KO cells (Figure 2B and I).

Next, we explored the role of WNT signaling. β-CATENIN was persistently active at D3 following *c-JUN* knockout (Figure 3J). Analysis of scRNA-seq data using CellChat (55) confirmed that WNT pathways were upregulated upon *c-JUN* knockout (Figure 3K). Altogether, these findings indicate a negative role for *c-JUN* in controlling mesoderm gene expression.

### Human *c-JUN* maintains mesoderm chromatin in a closed state

**Figure 4.**
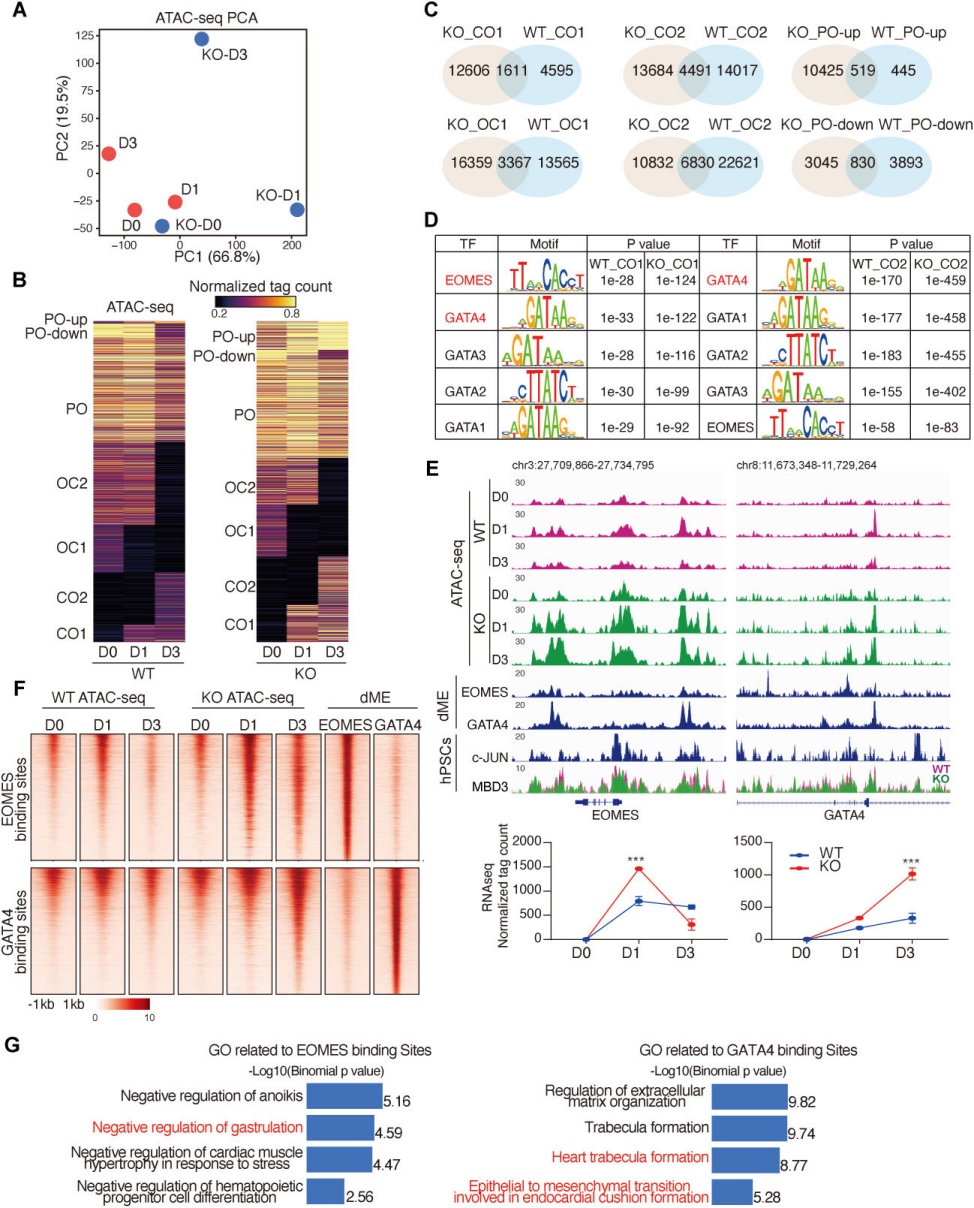
*c-JUN* inhibits mesoderm chromatin remodeling. (**A**) PCA of the ATAC-seq data (HRA003227) (28) showing alternate mesoderm development trajectories for WT and *c-JUN* KO cells. (**B**) Loci of open chromatin were arranged into CO/OC/PO categories. CO: loci from close to open, OC: loci from open to close and PO: permanently open. PO was subdivided into PO-down (that remained open but decreased), PO-up (that remained open and increased) and PO (that remained open and unchanged). (**C**) Venn plots for the number of peaks for overlapping between each dependent class, derived from panel (B). (**D**) TF motif analysis for ATAC-seq peaks of CO/OC categories between WT and *c-JUN* KO cells. (**E**) Genome view of the ATAC-seq data during hPSC (D0) differentiated into mesoderm cell (D3), and EOMES (GSM1505630, GSM1505631) (3), GATA4 (GSM1505644, GSM1505645) (3) ChIP-seq data in dME and c-JUN, MBD CUT&RUN data in hPSC on select target genes. The corresponding gene expression data were below the genome view. (**F**) Heatmap shows the signal of WT and *c-JUN* KO ATAC-seq data during hPSC (D0) differentiated into mesoderm cell (D3), and EOMES (GSM1505630, GSM1505631) (3), GATA4 (GSM1505644, GSM1505645) (3) ChIP-seq data in dME on EOMES and GATA4 binding sites. (**G**) GO analyzed by GREAT (56) and showing the different biological functions of *EOMES* and *GATA4* regulated genes.

To study how *c-JUN* influences chromatin remodeling during the transition from hPSCs to mesoderm, we analyzed chromatin accessibility data (ATAC-seq); PCA shows the distinct trajectory for hPSCs to mesoderm with and without *c-JUN* (Figure 4A). We then classified ATAC-seq peaks into three categories: CO (closed and then opened), OC (open and then closed) and PO (persistently open), employing a strategy similar to our previous study (Figure 4B and C) (26). The CO and OC peaks were further divided into two subgroups (CO1-2 and OC1-2), based on the day of chromatin opening and closing during the transition from hPSCs to mesoderm. Comparing the OC/CO loci between WT and *c-JUN* KO data, we found the shared open and closed loci were fewer than the loci that were uniquely open or closed, indicating that knocking out *c-JUN* lead to a distinct chromatin assembling pattern during the hPSCs-to-mesoderm transition (Figure 4C).

Motif analysis (57) revealed that OC loci were enriched with pluripotent TFs, including SOX and OCT, marking the exit from pluripotency (Supplementary Figure S4B and C). Conversely, CO loci were enriched with pivotal mesoderm-associated TFs such as those from the GATA family (Supplementary Figure S4B and C). Notably, *EOMES/GATA4* were significantly enriched in *c-JUN* KO CO loci (Figure 4D). Additionally, the chromatin accessibility of *EOMES* and *GATA4* loci increased when *c-JUN* was knocked out during the differentiation of hPSCs to the mesoderm, accompanied by upregulation of their gene expression (Figure 4E). Altogether, these findings suggest enhanced activity of these TFs upon *c-JUN* knockout.

As an early response TF, EOMES (3) dimerizes and can co-bind with GATA4, potentially contributing to the activation of *GATA4* expression. Interestingly, MBD3 also binds to the *EOMES* and *GATA4* loci, with a slight decline in some loci upon *c-JUN* knockout in hPSCs, suggesting that *c-JUN* may directly regulate the chromatin remodeling of *EOMES* and *GATA4* (Figure 4E). Next, we examined the binding profiles of *EOMES* and *GATA4* at the mesoderm stage (3). *EOMES* was enriched at CO1 and CO2 loci; however, *GATA4* was enriched at CO2 loci in *c-JUN* KO cells (Supplementary Figure S4D). Intriguingly, the binding sites of *EOMES* and *GATA4* did not overlap (Figure 4F). Consistent with motif analysis, the accessibility of *EOMES* binding sites increased at D1 in *c-JUN* KO cells, aligning with their roles in regulating primitive streak formation and gastrulation (58,59) (Figure 4F and G). Moreover, *GATA4* binding sites, crucial for regulating epithelial-to-mesenchymal transition and heart trabecula formation (60,61), displayed enhanced accessibility during mesoderm formation upon *c-JUN* deletion (Figure 4F and G). In conclusion, these data suggest that knocking out *c-JUN* enhances *EOMES* activity to induce gastrulation in the early stage, with *GATA4* subsequently directing the establishment of mesoderm chromatin signatures.

### *MBD3* knockdown enhances hPSCs differentiation into *PDGFRA*+ mesoderm cells

**Figure 5.**
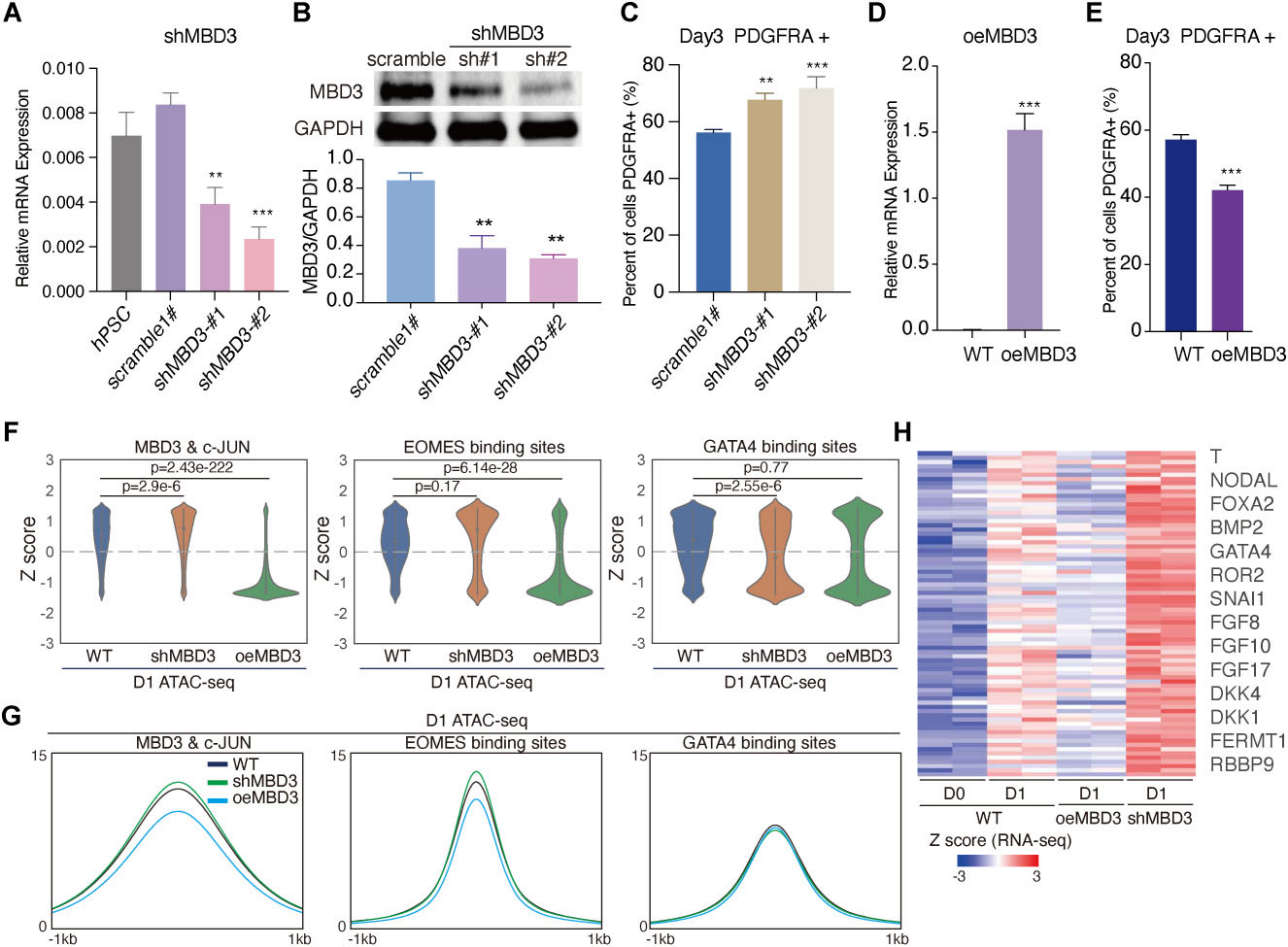
*MBD3* regulates mesoderm-associated chromatin open during pluripotency-to-mesoderm transition. (**A**) qRT-PCR detects *MBD3* knockdown efficiency in WT hPSC. Here, we picked up two shRNAs (sh*MBD3*-#1 and sh*MBD3*-#2) that could significantly downregulate *MBD3* mRNA level, and scramble shRNA as a control. Data were from four biological replicates in two independent experiments. Data are shown as the mean ± SEM. **P-value < 0.01, ***P-value < 0.001, one-way ANOVA with Sidak correction between the scramble groups. (**B**) Western blot showing the protein level of MBD3 in scramble and two *MBD3* knockdown cell lines (sh*MBD3*-#1 and sh*MBD3*-#2). Data were from three biological replicates—here, we show one of them. The lower bar plot shows the quantitation of the MBD3 protein level. Data are shown as the mean ± SEM. **P-value < 0.01, one-way ANOVA with Sidak correction between the scramble and *MBD3* knockdown groups (sh*MBD3*-#1 and sh*MBD3*-#2). (**C**) Bar chart summarizing the FACS analysis at day 3 of *PDGFRA*+ mesoderm cells between scramble and two *MBD3* knockdown cell lines (sh*MBD3*-#1 and sh*MBD3*-#2). Data were from nine biological replicates in three independent experiments. Data are shown as the mean ± SEM. **P-value < 0.01, ***P-value < 0.001, one-way ANOVA with Sidak correction between the scramble and *MBD3* knockdown groups (sh*MBD3*-#1 and sh*MBD3*-#2). (**D**) Bar chart shows *MBD3* mRNA level when *MBD3* over overexpressed in WT hPSCs (named oe*MBD3*). Data were from six biological replicates in two independent experiments. Data are shown as the mean ± SEM. ***P-value < 0.001, unpaired *t*-test between the WT and oe*MBD3* cells. (**E**) Bar chart summarizing the FACS analysis at day 3 of *PDGFRA*+ mesoderm cells between WT and oe*MBD3* cell lines. Data were from 14 biological replicates in four independent experiments. Data are shown as the mean ± SEM. ***P-value < 0.001, unpaired *t*-test between the WT and oe*MBD3* cells. (**F**) The violin plot revealed that knockdown *MBD3* increased the accessibility of MBD3 & c-JUN co-binding loci, as well as EOMES binding sites on day 1 (D1). In contrast, overexpression of *MBD3* (*oeMBD3*) decreased the accessibility of these loci, while having no significant effect on GATA4 binding sites. (**G**) Average ATAC-seq read density of D1 WT, sh*MBD3* and oe*MBD3* cells during inducting hPSC differentiation into mesoderm, the pileups are centered on MBD3 & c-JUN co-binding loci, EOMES binding sites and GATA4 binding sites. (**H**) Heatmap showed that knockdown *MBD3* enhanced 77 genes higher expression than WT in day 1 (D1). Conversely, oe*MBD3* inhibits the expression of these genes.

c-JUN interacts with MBD3, but it remains unclear whether *MBD3* influences mesoderm differentiation. To explore this, we generated two shRNAs to knockdown *MBD3* in hPSCs (Figure 5A and B). Consistent with our hypothesis, *MBD3* knockdown enhanced mesoderm generation (Figure 5C). Interestingly, overexpression of *MBD3* in hPSCs impaired mesoderm differentiation (Figure 5D and E, and Supplementary Figure S5A and B). These data indicate that the *MBD3*–NuRD complex is a key regulator, working alongside factors like *c-JUN* to modulate hPSC differentiation into mesoderm.

Given the potential role of *MBD3* in chromatin remodeling, we assessed chromatin accessibility following *MBD3* knockdown or overexpression. Notably, *MBD3* knockdown slightly enhanced chromatin accessibility of MBD3 & c-JUN co-bound loci (Figure 5F and G). Conversely, *MBD3* overexpression repressed MBD3 & c-JUN co-bound loci and *EOMES* binding sites on day 1 (D1) (Figure 5F and G). Interestingly, *GATA4* binding sites were not affected by *MBD3* overexpression (Figure 5F and G), possibly because *GATA4* is less active than *EOMES* in the early stage (D1) (Figure 4E). Consistent with these chromatin accessibility dynamics, *MBD3* knockdown upregulated the expression of key genes involved in mesoderm formation, such as *T*, *NODAL*, *FOXA2*, *BMP2*, *DKK1* and *SNAI1* (Figure 5H, and Supplementary Figure S5C). In contrast, these genes were repressed when *MBD3* was overexpressed (Figure 5H). In summary, these data suggest that the *MBD3*–NuRD complex regulates mesoderm cell fate by remodeling mesoderm-specific chromatin.

### Overexpression of *c-JUN* directs cell fate toward a fibroblast-like state

While we have shown that knocking out *c-JUN* enhances mesoderm development, we were curious about the impact of elevated *c-JUN* expression on cell fate. To explore this, we generated an inducible hPSCs line by introducing the pb-TetOn-*c-JUN* plasmid into *c-JUN* KO hPSCs (Supplementary Figure S6A). Notably, *c-JUN* remained inactive without dox treatment but was robustly expressed under 1 μg/ml dox treated (Supplementary Figure S6B and C). Critically, TetOn *c-JUN* hPSCs exhibited similar differentiation efficiency in the absence of dox, confirming that the TetOn *c-JUN* plasmid did not alter the *c-JUN* KO hPSCs state (Supplementary Figure S6D). We refer to these cells treated with 1 μg/ml dox as ‘OE’. Surprisingly, key mesoderm factors *EOMES* and *GATA4* were downregulated in OE *c-JUN* cells during differentiation into mesoderm (Supplementary Figure S6E and F)

**Figure 6.**
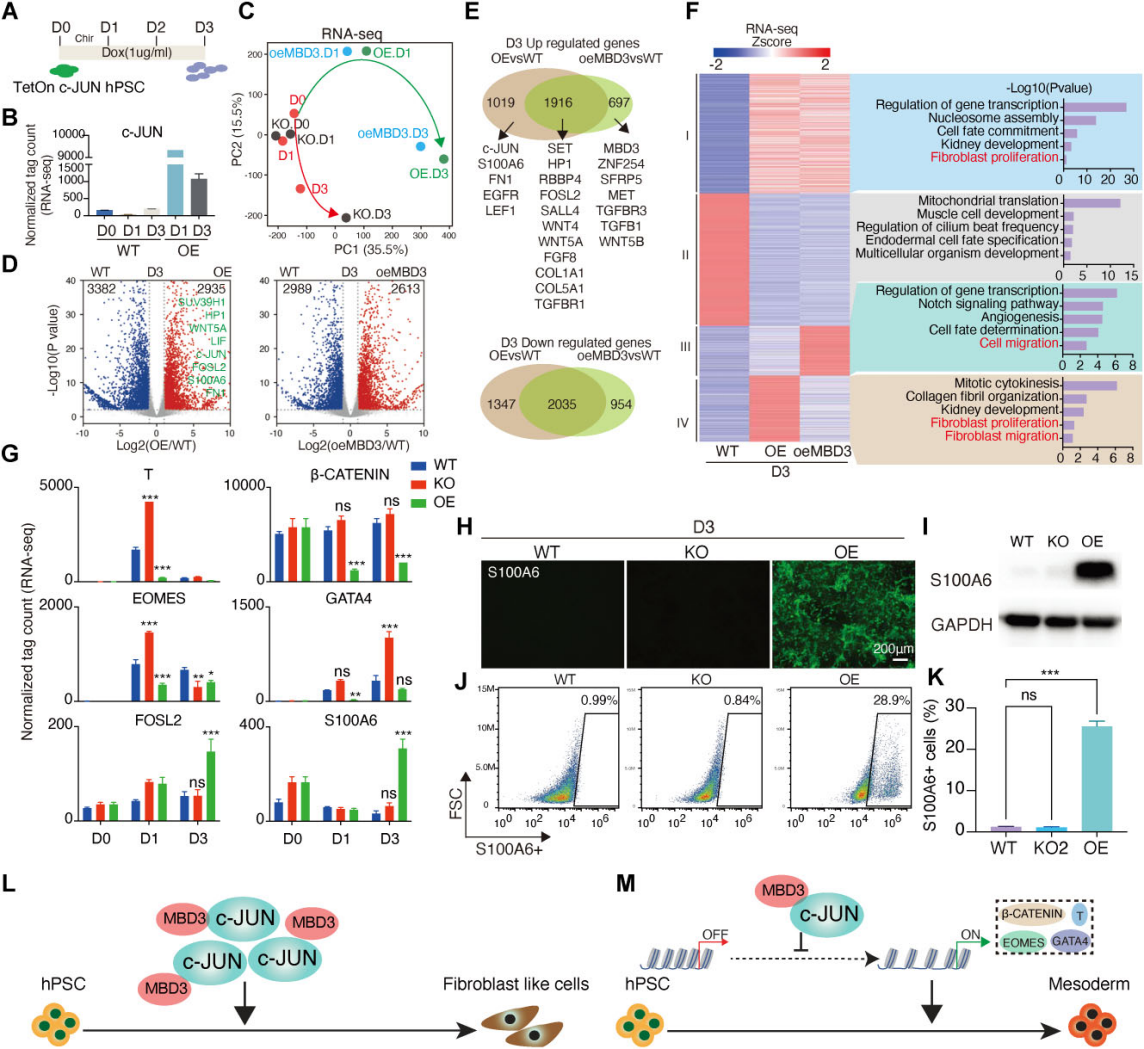
Overexpression of *c-JUN* directing hPSC into fibroblast-like cells. (**A**) Dox (1 μg/ml) was added into mesoderm induction medium from D0 to D3 to induce *c-JUN* OE. (**B**) The mRNA level of c-JUN in WT and *c-JUN* OE cell line during hPSC-to-mesoderm transition. (**C**) PCA shows that overexpressed *c-JUN* or *MBD3* directs cell fate into a new branch during mesoderm induction. (**D**) Volcano plot shows DEGs between WT and *c-JUN* OE cells (OE) or *MBD3* OE cells (oe*MBD3*) in D3. D = day. (**E**) Venn plot shows the up and downregulated genes (defined as DEGs in panel D) in WT, *c-JUN* OE and *MBD3* OE conditions. (**F**) Heatmap shows the four group DEGs in WT, *c-JUN* OE and *MBD3* OE conditions. GO was analyzed by DAVID website and shows the different biological functions of the four group DEGs. (**G**) Bar chart shows the RNA-seq data of selected target genes. Data were from two biological replicates and are shown as the mean ± SEM. ***P-value < 0.001, **P-value < 0.01, two-way ANOVA multiple comparisons between the WT control and *c-JUN* KO or *c-JUN* OE groups. (**H**) Immunostaining shows that OE *c-JUN* promotes fibroblast-like cell marker S100A6 expression. Scale bar, 200 μm. (**I**) Western blot shows that OE *c-JUN* promotes fibroblast-like cell marker S100A6 expression in D3. (**J**) Flow cytometry quantification of the percent of *S100A6*+ cells in 3-day induction in WT, KO (*c-JUN* KO) and OE (*c-JUN* OE) groups. Here, we use *S100A6* as the marker of fibroblast cells. (**K**) Flow cytometry quantification of fibroblast-like cells’ differentiation efficiency in D3. Overexpression of *c-JUN* enhances *S100A6*+ fibroblast-like cells during 3 days of induction. Data were from three biological replicates and are shown as the mean ± SEM. ***P-value < 0.001, one-way ANOVA with Sidak correction between the WT and KO (*c-JUN* KO) or OE (*c-JUN* OE) groups. (**L**) The schematic illustrates that high expression levels of *c-JUN* or *MBD3* can guide hPSCs toward a fibroblast-like state. (**M**) Schematic for the role of *c-JUN* in remodeling chromatin remodeling during hPSC-to-mesoderm transition. By inhibiting the opening of mesoderm-specific chromatin, c-JUN represses the activation of *β-CATENIN, T, EOMES* and *GATA4*, consequently leading to reduced efficiency in mesoderm development.

To uncover the specific cell fate triggered by *c-JUN* overexpression, we analyzed RNA-seq data from OE *c-JUN* cells (Figure 6A and B). Strikingly, OE *c-JUN* induced a unique trajectory that did not cluster with WT or c*-JUN* KO data (Figure 6C, and Supplementary Figure S6G). Interestingly, as we demonstrated in Figure 3, *c-JUN* interacts with *MBD3* to restrict mesoderm chromatin remodeling. We found that overexpressing *MBD3* (oe*MBD3*) leads to a cell fate trajectory similar to that observed with OE *c-JUN* (Figure 6C, and Supplementary Figure S6G). A comparison of up and downregulated genes (D3) among WT, *c-JUN* OE (OE) and *MBD3* OE (oe*MBD3*) datasets revealed an intriguing pattern: both *c-JUN* OE and *MBD3* OE resulted in 1916 commonly upregulated genes and the downregulation of over 2000 common genes (Figure 6D and E). Notably, the shared upregulated genes include *SET*, *HP1* and *SALL4*—known for their roles in nucleosome and heterochromatin assembly as well as *FOSL2*, *WNT4*, *WNT5A*, *FGF8* and *COL1A1*, which are associated with fibroblast cell identify (Figure 6E). Furthermore, clustering the DEGs of WT, *c-JUN* OE and *MBD3* OE datasets into four groups, followed by GO analysis, indicates that both *c-JUN* OE and *MBD3* OE co-upregulate fibroblast-related genes. Additionally, *c-JUN* OE specifically increased the expression of fibroblast-related genes, such as *S100A6*, *FN1*, *EGFR* and *LEF1* (Figure 6E and F). Those findings suggest that high levels of *c-JUN* may cooperate with *MBD3* to suppress mesoderm cell fate while promoting a fibroblast-like cell fate (Figure 6F, and Supplementary Figure S6H).

To further investigate whether c-JUN directs hPSCs toward a fibroblast-like cell fate, we observed significant repression of *β-CATENIN* and *T* expression, two factors essential for initiating mesoderm differentiation (10,13,62,63) (Figure 6G). Notably, *EOMES* and *GATA4*, which were upregulated by *c-JUN* KO, were significantly downregulated by *c-JUN* OE (Figure 6G). However, the gene expression and protein levels of the fibroblast marker gene S100A6 were activated in D3 upon *c-JUN* OE (Figure 6G–I). FACS assay revealed the proportion of *S100A6*+ fibroblast-like cells increased from 0.99% to 28.9% when *c-JUN* was overexpressed (Figure 6J and K). Collectively, these findings demonstrate that heightened *c-JUN* expression requires MDB3 to induce the differentiation of hPSC into a fibroblast-like cell fate (Figure 6L and M).

## Discussion

Early embryonic development is a crucial event for human body formation. Here, we show that *c-JUN*, a member of the AP-1 family, plays a negative role in regulating mesoderm cell fate. Our data suggest that *c-JUN* can directly interact with *MBD3*, a component of the NuRD complex, to constrain the opening of mesoderm-specific chromatin, thereby repressing *EOMES* and *GATA4* activation, which are crucial for regulating mesoderm cell fate during hPSC-to-mesoderm transition (Figure 6M). Three decades ago, *c-JUN* was identified as essential for mouse embryonic development, with deletion of *c-JUN* leading to embryonic lethality at ∼E13.5, primarily due to impaired hematogenesis and liver development. Chimeric complementation studies revealed that knocking out *c-JUN* inhibits the differentiation of mESC into liver cells, without affecting heart development (21). However, the distinct evolutionary paths and environmental adaptations of mice and humans have led to significant differences between the two species, suggesting that the same genes may play varied roles in their respective development processes (64,65).

*c-JUN* and *MBD3*–NuRD complex are two factors that have opposite effects on cellular reprogramming. *c-JUN* is a TF that inhibits the conversion of somatic cells into iPSCs by activating mesenchymal genes and repressing pluripotent genes (23,66). *MBD3* is a component of the NuRD complex, which is a co-repressor that mediates gene silencing through histone deacetylation and chromatin remodeling (67,68). *MBD3* is required for the development of pluripotent cells *in vivo* and *in vitro* (68). Interestingly, c-JUN can interact with MBD3 and recruit the NuRD complex to AP-1 target genes, resulting in gene repression (20,69). However, this interaction is disrupted by *c-JUN* N-terminal phosphorylation, which is induced by JNK signaling (20,69). Therefore, the *c-JUN* and *MBD3*–NuRD complex have a dynamic and context-dependent relationship that regulates cell fate decisions (70).

WNT signaling plays a pivotal role in embryonic development, orchestrating diverse cellular processes throughout embryogenesis (71). This intricate pathway is indispensable for specifying cell fate within the mesoderm, including mesodermal cardiac progenitor cells, and cardiac development (72–74). Our previous research indicates that knockout of *c-JUN* enhances cardiac generation, whereas its overexpression hampers this process (28). In this study, we delve deeper into the role of *c-JUN* in regulating the mesoderm, which is an early and crucial stage during cardiogenesis. We observed a swift degradation of *c-JUN* post CHIR treatment, concomitant with a decrease in mRNA levels (29). Notable, deleting *c-JUN* leads to upregulation of *β-CATENIN* expression, thereby enhancing WNT signaling and expediting mesoderm formation. Conversely, elevated *c-JUN* expression suppresses *β-CATENIN* and steers cell fate toward a fibroblast-like state. Consequently, *c-JUN* acts as an antagonist to WNT signaling during the transition of hPSC to the mesoderm stage.

Understanding the molecular mechanisms underlying *c-JUN*-mediated regulation of cell fate decisions in humans is of great importance for both basic biology and clinical applications. Elucidating the downstream targets and signaling pathways controlled by *c-JUN* will provide valuable insights into the cellular processes involved in development, tissue regeneration and disease progression. Furthermore, targeting *c-JUN* and its associated pathways may open up new avenues for therapeutic interventions in various diseases, including cancer and regenerative medicine.

## Supplementary Material

gkaf001_Supplemental_File

## Data Availability

*c-JUN* and *MBD3*-related raw sequence data generated in this study including ATAC-seq, RNA-seq and CUT&RUN data were deposited in the Genome Sequence Archive (Genomics, Proteomics & Bioinformatics 2021) in the National Genomics Data Center (Nucleic Acids Research 2021), China National Center for Bioinformation/Beijing Institute of Genomics, Chinese Academy of Sciences (GSA: HRA006991) and are publicly accessible at https://ngdc.cncb.ac.cn/gsa. WT and *c-JUN* KO-related scRNA-seq, bulk RNA-seq, ATAC-seq and CUT&RUN are accessible at https://ngdc.cncb.ac.cn/gsa (GSA: HRA003227). The mass spectrometry proteomics data have been deposited to the ProteomeXchange (PXD048153) and are publicly accessible at https://proteomecentral.proteomexchange.org/cgi/GetDataset?ID=PXD048153.
