# Supplementary material for: c-JUN: a chromatin repressor that limits mesoderm differentiation in human pluripotent stem cells
Source: Nucleic Acids Res. 2025 Jan 29;53(3):gkaf001. doi: 10.1093/nar/gkaf001 (PMC11760979; doi:10.1093/nar/gkaf001)
Supplement: gkaf001_Supplemental_File [file gkaf001_supplemental_file.pdf]

Figure.S1

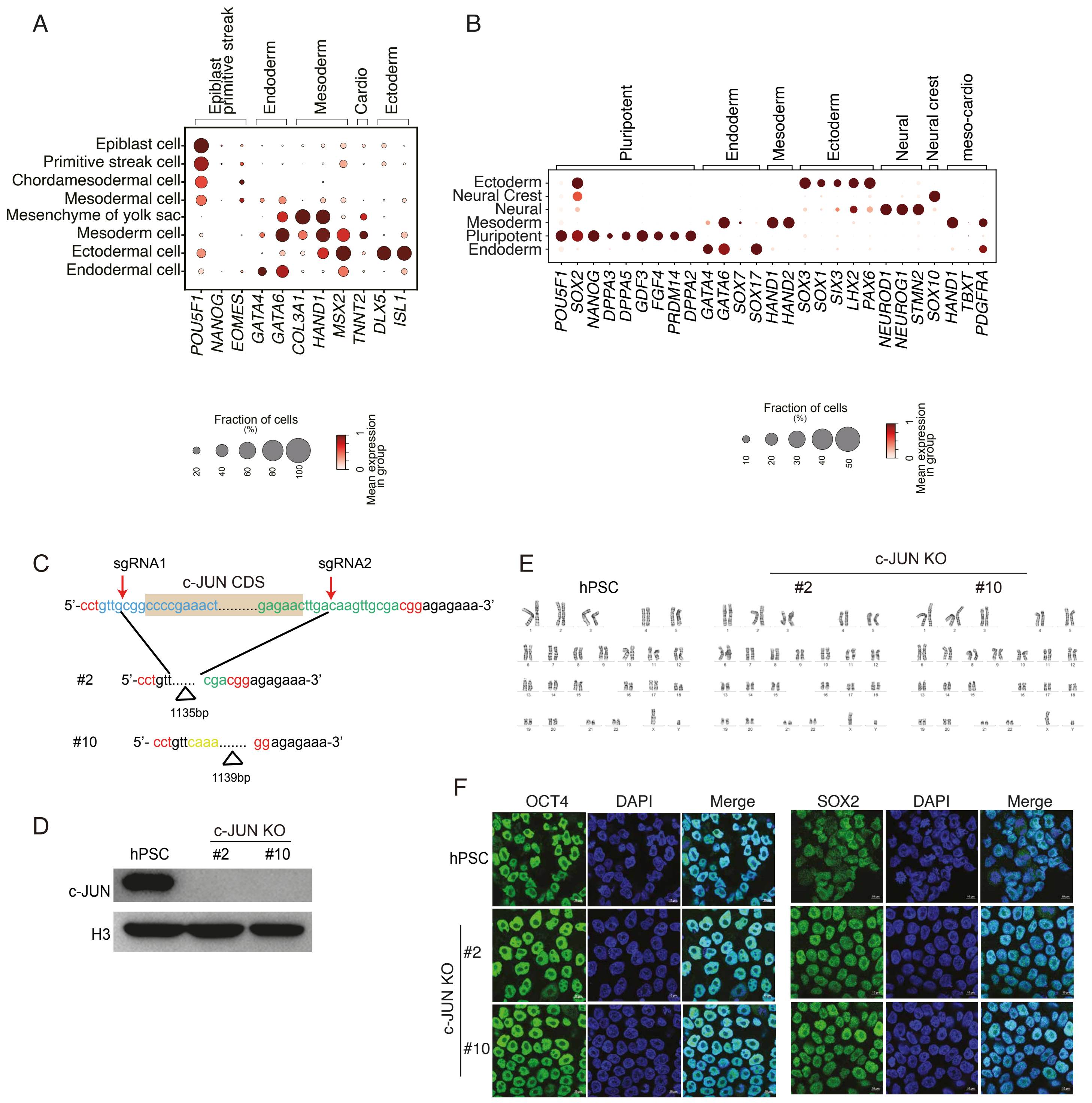

A
Epiblast primitive streak
Endoderm
Mesoderm
Cardio
Ectoderm
Epiblast cell
Primitive streak cell
Chordamesodermal cell
Mesodermal cell
Mesenchyme of yolk sac
Mesoderm cell
Ectodermal cell
Endodermal cell
POU5F1
NANOG
EOMES
GATA4
GATA6
COL3A1
HAND1
MSX2
TNNT2
DLX5
ISL1
Fraction of cells (%)
20
40
60
80
100
Mean expression in group
0
1
B
Pluripotent
Endoderm
Mesoderm
Ectoderm
Neural
Neural crest
meso-cardio
Ectoderm
Neural Crest
Neural
Mesoderm
Pluripotent
Endoderm
POU5F1
SOX2
NANOG
DPPA3
DPPA5
GDF3
FGF4
PRDM14
DPPA2
GATA4
GATA6
SOX7
SOX17
HAND1
HAND2
SOX3
SOX1
SIX3
LHX2
PAX6
NEUROD1
NEUROG1
STMN2
SOX10
HAND1
TBXT
PDGFRA
Fraction of cells (%)
10
20
30
40
50
Mean expression in group
0
1
C
sgRNA1
sgRNA2
c-JUN CDS
5'-cctgttgcggccccgaaact..........gagaacttgacaagttgcgacggagagaaa-3'
#2
5'-cctgtt...... cgacggagagaaa-3'
1135bp
#10
5'- cctgttcaaa....... ggagagaaa-3'
1139bp
D
c-JUN KO
hPSC
#2
#10
c-JUN
H3
E
c-JUN KO
hPSC
#2
#10
F
OCT4
DAPI
Merge
SOX2
DAPI
Merge
hPSC
c-JUN KO
#2
#10

Figure.S1 Knock out *c-JUN* does not affect pluripotency

A. Dotplots showing the expression level and percentage of cells expressing the indicated genes in the cells from the gastrulating human embryo cells (48). Marker genes for the indicated lineages are labeled across the top of the plot, cell types are indicated on the left-hand side. Cell-type designation labels are taken from the original study.

B. Dotplots showing the expression level and percentage of cells expressing the indicated genes in the cells from the embryoid bodies (49). Marker genes for the indicated lineages are indicated across the top of the plot, cell types are indicated on the left-hand side.

C. Strategy to knock-out *c-JUN* by CRISPR/Cas9. Two sgRNA were targeted at both ends of the *c-JUN* coding region. *c-JUN* knock-out clones were sequenced to validate that *c-JUN* protein-coding regions were deleted.

D. Western blot shows the c-JUN not expressed in *c-JUN* knock-out clones.

E. hPSC and *c-JUN* knock-out clones have normal karyotypes.

F. Immunostaining shows knock-out of *c-JUN* does not influence pluripotent genes OCT4 and SOX2 protein expression. Scale bar, 10μm.

Figure.S2

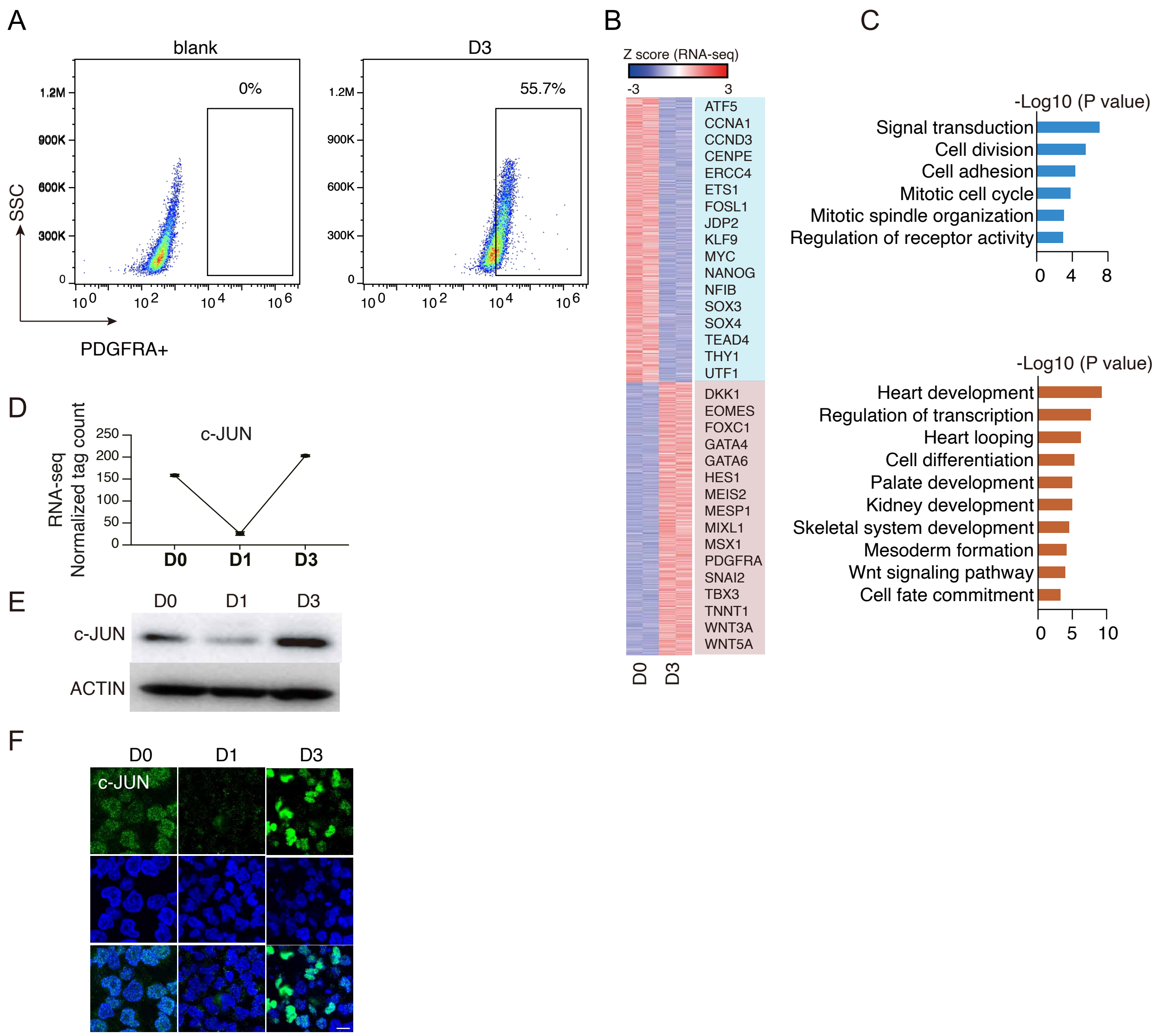

A
blank
0%
D3
55.7%
1.2M
900K
600K
300K
0
SSC
PDGFRA+
B
Z score (RNA-seq)
-3
3
ATF5
CCNA1
CCND3
CENPE
ERCC4
ETS1
FOSL1
JDP2
KLF9
MYC
NANOG
NFIB
SOX3
SOX4
TEAD4
THY1
UTF1
DKK1
EOMES
FOXC1
GATA4
GATA6
HES1
MEIS2
MESP1
MIXL1
MSX1
PDGFRA
SNAI2
TBX3
TNNT1
WNT3A
WNT5A
D0
D3
C
-Log10 (P value)
Signal transduction
Cell division
Cell adhesion
Mitotic cell cycle
Mitotic spindle organization
Regulation of receptor activity
0
4
8
-Log10 (P value)
Heart development
Regulation of transcription
Heart looping
Cell differentiation
Palate development
Kidney development
Skeletal system development
Mesoderm formation
Wnt signaling pathway
Cell fate commitment
0
5
10
D
c-JUN
RNA-seq
Normalized tag count
250
200
150
100
50
0
D0
D1
D3
E
D0
D1
D3
c-JUN
ACTIN
F
D0
D1
D3
c-JUN

Figure.S2 Differentiation hPSC into the mesoderm

A. Flow cytometry quantification of the percentage of *PDGFRA*+ cells in three days of induction. Here we use *PDGFRA* as the marker of Mesoderm cells.

B. Heatmap shows the differential expression genes (DEGs) of pluripotent hPSC (D0) and differentiated Mesoderm cells (D3).

C. Gene ontology (GO) analysis shows the different biological functions of the DEGs in **B**.

D. RNA-seq data shows *c-JUN* expression in hPSC (D0), D1 and D3 cells.

E. Western blot shows the expression of c-JUN protein in hPSC (D0), D1 and D3 cells.

F. Immunostaining shows the expression of c-JUN in hPSC (D0), D1 and D3 cells. Scale bar, 10μm.

Figure.S3

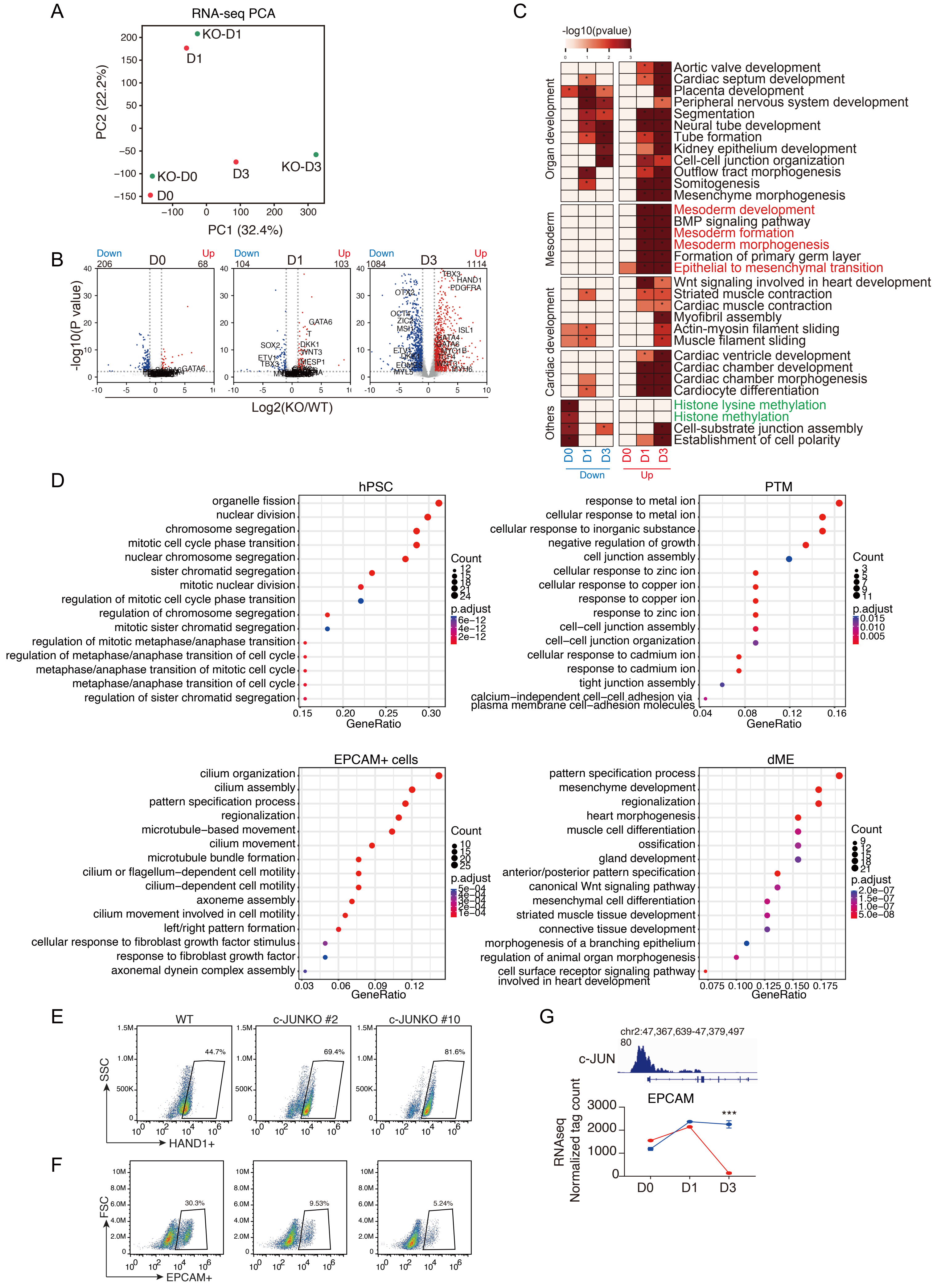
A
RNA-seq PCA
KO-D1
D1
KO-D0
D0
D3
KO-D3
PC1 (32.4%)
PC2 (22.2%)
B
Down
Up
D0
206
68
D1
104
103
D3
1084
1114
-log10(P value)
Log2(KO/WT)
GATA6
SOX2
ETV1
TBX3
DKK1
WNT3
MESP1
OTX2
OCT4
ZIC2
MSI1
TBX3
HAND1
PDGFRA
ISL1
GATA4
GATA6
MYO1B
MYL5
MYH6
C
-log10(pvalue)
Organ development
Mesoderm
Cardiac development
Others
Aortic valve development
Cardiac septum development
Placenta development
Peripheral nervous system development
Segmentation
Neural tube development
Tube formation
Kidney epithelium development
Cell-cell junction organization
Outflow tract morphogenesis
Somitogenesis
Mesenchyme morphogenesis
Mesoderm development
BMP signaling pathway
Mesoderm formation
Mesoderm morphogenesis
Formation of primary germ layer
Epithelial to mesenchymal transition
Wnt signaling involved in heart development
Striated muscle contraction
Cardiac muscle contraction
Myofibril assembly
Actin-myosin filament sliding
Muscle filament sliding
Cardiac ventricle development
Cardiac chamber development
Cardiac chamber morphogenesis
Cardiocyte differentiation
Histone lysine methylation
Histone methylation
Cell-substrate junction assembly
Establishment of cell polarity
D0 D1 D3 D0 D1 D3
Down
Up
D
hPSC
organelle fission
nuclear division
chromosome segregation
mitotic cell cycle phase transition
nuclear chromosome segregation
sister chromatid segregation
mitotic nuclear division
regulation of mitotic cell cycle phase transition
regulation of chromosome segregation
mitotic sister chromatid segregation
regulation of mitotic metaphase/anaphase transition
regulation of metaphase/anaphase transition of cell cycle
metaphase/anaphase transition of mitotic cell cycle
metaphase/anaphase transition of cell cycle
regulation of sister chromatid segregation
GeneRatio
Count
p.adjust
PTM
response to metal ion
cellular response to metal ion
cellular response to inorganic substance
negative regulation of growth
cell junction assembly
cellular response to zinc ion
cellular response to copper ion
response to copper ion
response to zinc ion
cell−cell junction assembly
cell−cell junction organization
cellular response to cadmium ion
response to cadmium ion
tight junction assembly
calcium−independent cell−cell adhesion via plasma membrane cell−adhesion molecules
GeneRatio
EPCAM+ cells
cilium organization
cilium assembly
pattern specification process
regionalization
microtubule−based movement
cilium movement
microtubule bundle formation
cilium or flagellum−dependent cell motility
cilium−dependent cell motility
axoneme assembly
cilium movement involved in cell motility
left/right pattern formation
cellular response to fibroblast growth factor stimulus
response to fibroblast growth factor
axonemal dynein complex assembly
GeneRatio
dME
pattern specification process
mesenchyme development
regionalization
heart morphogenesis
muscle cell differentiation
ossification
gland development
anterior/posterior pattern specification
canonical Wnt signaling pathway
mesenchymal cell differentiation
striated muscle tissue development
connective tissue development
morphogenesis of a branching epithelium
regulation of animal organ morphogenesis
cell surface receptor signaling pathway involved in heart development
GeneRatio
E
WT
c-JUNKO #2
c-JUNKO #10
44.7%
69.4%
81.6%
SSC
HAND1+
F
30.3%
9.53%
5.24%
FSC
EPCAM+
G
chr2:47,367,639-47,379,497
c-JUN
EPCAM
***
RNAseq
Normalized tag count
D0
D1
D3

Figure.S3 *c-JUN* inhibits mesoderm transcriptome activation

A. Principal Component Analysis (PCA) of the RNA-seq data showing the dynamics of the transcriptome of WT and *c-JUN* KO cells during hPSC differentiate into Mesoderm cells.

B. Volcano plot for DEGs in hPSC (D0), D1 and D3 between WT and *c-JUN* KO cells. Red dots represent significantly highly expressed genes in WT, blue dots represent significantly highly expressed genes in KO cells, and grey dots indicate non-significance of difference in gene expression between WT and *c-JUN* KO cells. Genes were considered significantly different if their q-value was < 0.05 and their absolute fold-change was > 2.0.

C. Gene ontology (GO) analysis for DEGs in (**B**). * Benjamini-Hochberg corrected p-value （Q value）< 0.05 from a Wallenius noncentral hypergeometric distribution implemented in geseq.

D. Gene ontology (GO) analysis for DEGs in each cell type defined from the scRNA-seq data.

E. Flow cytometry quantification of mesoderm differentiation efficiency by detecting the expression of mesoderm marker gene *HAND1*.

F. Flow cytometry quantification of mesoderm differentiation efficiency by detecting the expression of *EPCAM*.

G. Genome view of c-JUN Cut-Run data in hPSCs. c-JUN was robustly binding to the *EPCAM* promoter. Corresponding gene expression data were below the genome view.

Figure.S4

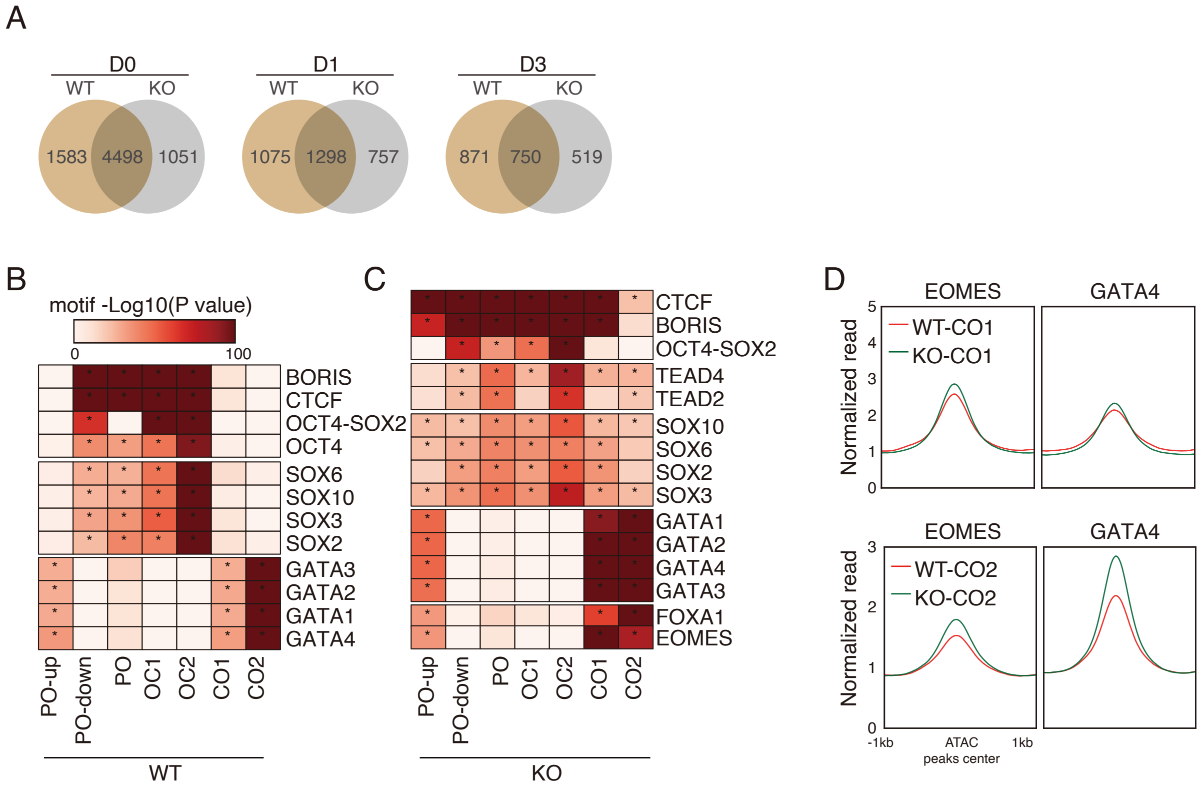
A
D0
WT
KO
1583
4498
1051
D1
WT
KO
1075
1298
757
D3
WT
KO
871
750
519
B
motif -Log10(P value)
0
100
BORIS
CTCF
OCT4-SOX2
OCT4
SOX6
SOX10
SOX3
SOX2
GATA3
GATA2
GATA1
GATA4
PO-up
PO-down
PO
OC1
OC2
CO1
CO2
WT
C
CTCF
BORIS
OCT4-SOX2
TEAD4
TEAD2
SOX10
SOX6
SOX2
SOX3
GATA1
GATA2
GATA4
GATA3
FOXA1
EOMES
PO-up
PO-down
PO
OC1
OC2
CO1
CO2
KO
D
EOMES
GATA4
Normalized read
WT-CO1
KO-CO1
WT-CO2
KO-CO2
-1kb
ATAC
peaks center
1kb

Figure.S4 Chromatin remodeling during hPSC to mesoderm transition

A. Venn diagrams showing the overlap genes binding by MBD3 in WT and KO conditions in D0 to D3. To indentify *MBD3* regulated genes, we applied a filter for peaks with a P value > 12 and subsequently selected target genes within a TSS with 5kb of an MBD3 peaks for each condition.

B. Motif analysis of peaks in CO/OC/PO categories of WT ATAC-seq data, CO/OC/PO peaks defined in **Figure 4B**.

C. Motif analysis of peaks in CO/OC/PO categories of *c-JUN* KO ATAC-seq data, CO/OC/PO peaks defined in **Figure 4B**.

D. Pileup of the mesoderm TFs EOMES (GSM1505630, GSM1505631) (3), GATA4 (GSM1505644, GSM1505645) (3) ChIP-seq data at the CO1 and CO2 ATAC-seq peaks in WT and *c-JUN* KO cells.

Figure.S5

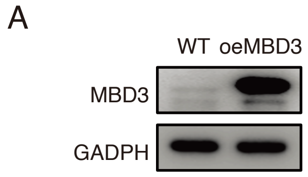
A
WT
oeMBD3
MBD3
GADPH

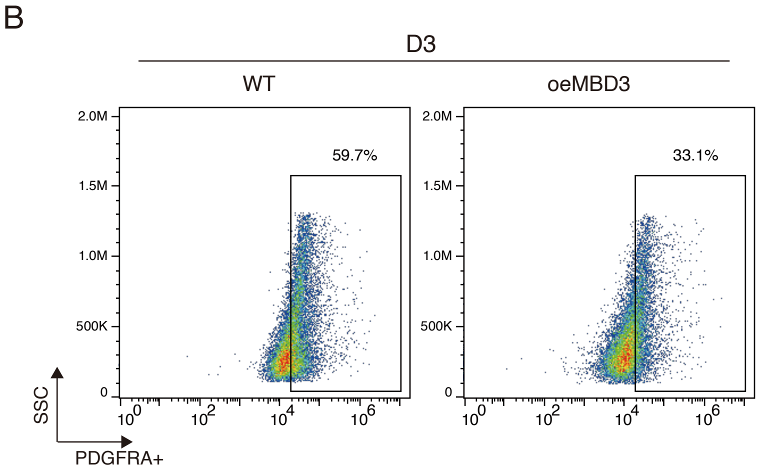
B
D3
WT
oeMBD3
59.7%
33.1%
2.0M
1.5M
1.0M
500K
0
SSC
PDGFRA+

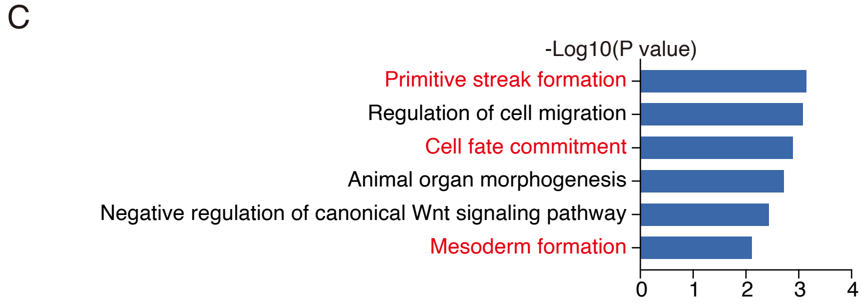
C
-Log10(P value)
Primitive streak formation
Regulation of cell migration
Cell fate commitment
Animal organ morphogenesis
Negative regulation of canonical Wnt signaling pathway
Mesoderm formation
0
1
2
3
4

Figure.S5 Over expression *MBD3* inhibits mesoderm formation

A. Western blot shows *MBD3* was overexpressed in WT hPSC.

B. Flow cytometry quantification of *PDGFRA*+ mesoderm differentiation efficiency between WT and oe*MBD3* cells.

C. Gene ontology (GO) analysis of the upregulated genes defined in **Figure 5H**.

Figure.S6

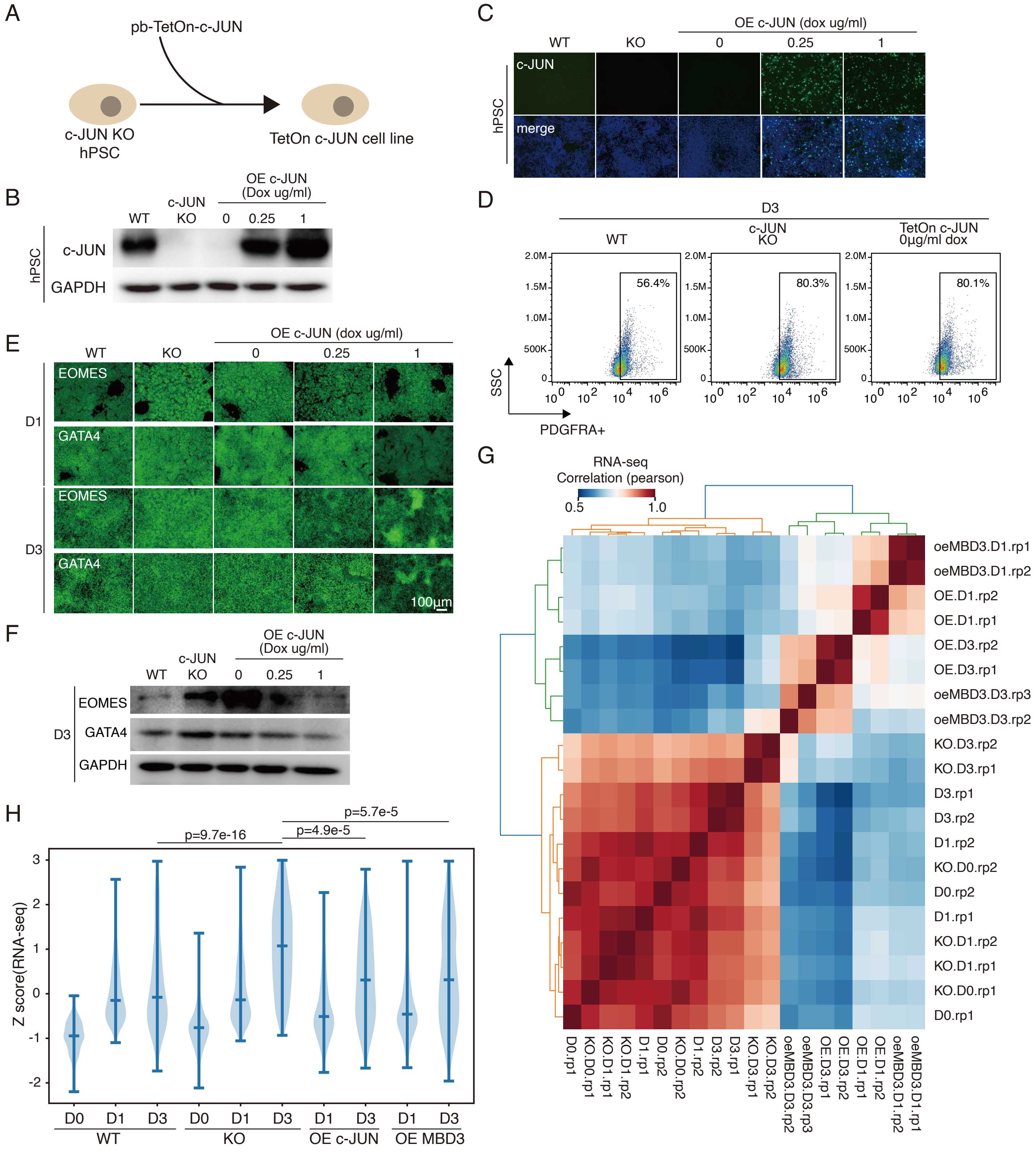
A
pb-TetOn-c-JUN
c-JUN KO
hPSC
TetOn c-JUN cell line
B
OE c-JUN
(Dox ug/ml)
WT
c-JUN KO
0
0.25
1
hPSC
c-JUN
GAPDH
C
OE c-JUN (dox ug/ml)
WT
KO
0
0.25
1
hPSC
c-JUN
merge
D
D3
WT
c-JUN KO
TetOn c-JUN 0μg/ml dox
56.4%
80.3%
80.1%
SSC
PDGFRA+
E
OE c-JUN (dox ug/ml)
WT
KO
0
0.25
1
D1
D3
EOMES
GATA4
100μm
F
OE c-JUN
(Dox ug/ml)
WT
c-JUN KO
0
0.25
1
D3
EOMES
GATA4
GAPDH
G
RNA-seq
Correlation (pearson)
0.5
1.0
oeMBD3.D1.rp1
oeMBD3.D1.rp2
OE.D1.rp2
OE.D1.rp1
OE.D3.rp2
OE.D3.rp1
oeMBD3.D3.rp3
oeMBD3.D3.rp2
KO.D3.rp2
KO.D3.rp1
D3.rp1
D3.rp2
D1.rp2
KO.D0.rp2
D0.rp2
D1.rp1
KO.D1.rp2
KO.D1.rp1
KO.D0.rp1
D0.rp1
H
p=9.7e-16
p=4.9e-5
p=5.7e-5
Z score(RNA-seq)
D0
D1
D3
WT
KO
OE c-JUN
OE MBD3

Figure.S6 Over expression *c-JUN* inhibits mesoderm formation

A. Schematic of built *c-JUN* overexpression cell line. *c-JUN* cod sequence was cloned into piggybac-TetOn plasmid which also contains rTTA and puromycin elements and then delivered into *c-JUN* KO hPSC by electroporator, and treated with puromycin to select TetOn *c-JUN* stable cell line.

B. Western blot shows c-JUN protein was induced under dox treatment. 1μg dox induced higher expression of c-JUN protein than 0.25μg dox and WT hPSC. And 1μg dox to induce *c-JUN* overexpression (OE *c-JUN*) was used for further research in this study.

C. Immunostaining shows c-JUN protein was induced under dox treatment, Scale bar, 100μm.

D. Flow cytometry quantification of the percent of *PDGFRA+* cells in three days induction. Without dox-treated TetOn *c-JUN* cell lines not affect *PDGFRA+* cell generation.

E. Immunostaining shows EOMES and GATA4 protein in WT, *c-JUN* KO, and *c-JUN* OE cells in D1 and D3. Scale bar, 100μm.

F. Western blot shows EOMES and GATA4 protein in WT, *c-JUN* KO, and 0, 0.25, 1μg/ml dox treated in D3.

G. Heatmap shows the correlation of RNA-seq data from WT, *c-JUN* KO, and *c-JUN* OE conditions.

H. Violin plot shows c-JUN and MBD3 co-binding genes were highly expressed in D3 when *c-JUN* was knocked out, while downregulation upon the ectopic expression of either *c-JUN* or *MBD3*.

Supplementary Table

qPCR primers		
**ID**	**Sequence (5'-3')**	**Description**
GAPDH-F	GAAGGTGAAGGTCGGAGTC	Reference gene fwd
GAPDH-R	GAAGATGGTGATGGGATTTC	Reference gene rev
OTX2-F	GTCACCAGCCATCTCAAT	Ectoderm marker gene *OTX2* fwd
OTX2-R	ATCCAAGCAGTCAGCATT	Ectoderm marker gene *OTX2* rev
PAX6-F	GTGTCCAACGGATGTGTGAG	Ectoderm marker gene *Pax6* fwd
PAX6-R	CTAGCCAGGTTGCGAAGAAC	Ectoderm marker gene *Pax6* rev
SNAI2-F	AAGGACACATTAGAACTCACA	Mesoderm marker gene *SNAI2* fwd
SNAI2-R	CTACACAGCAGCCAGATT	Mesoderm marker gene *SNAI2* rev
GATA4-F	CTCAGTCAGTGCGATGTC	Mesoderm marker gene *GATA4* fwd
GATA4-R	CAGGCTGTTCCAAGAGTC	Mesoderm marker gene *GATA4* rev
SOX17-F	CGCACGGAATTTGAACAG	Endoderm marker gene *SOX17* fwd
SOX17-R	CGTCAGGATAGTTGCAGTAA	Endoderm marker gene *SOX17* rev
CXCR4-F	GTGAGGCAGATGACAGATAT	Endoderm marker gene *CXCR4* fwd
CXCR4-R	ACAATACCAGGCAGGATAAG	Endoderm marker gene *CXCR4* rev
MBD3-F	CTGAGCACCTTCGACTTCCG	Human *MBD3* fwd
MBD3-R	CCGGCTGCTTGAAGATGGA	Human *MBD3* rev
**InFusion cloning primers**		
**ID**	**Sequence (5'-3')**	**Description**
c-JUN-PF1	TACCCTCGTAAAGAATTCATGACTGCAAAGATGGAAACGACCTTCTATGACGATG	Human *c-JUN* fwd (PB-TRE3G-*c-JUN*-SV40 polyA cloning)
c-JUN-PR1	AATAAACAAGTTACGCGTTCAAAATGTTTGCAACTGCTGCGTTAGCATGAGTTGGCA	Human *c-JUN* rev (PB-TRE3G-*c-JUN*-SV40 polyA cloning)
c-JUN-PF2	CTTCTTTTTCCTACAGCTCGAGATGACTGCAAAGATGGAAACGACCTTCT	Human *c-JUN* fwd (PB-CAG-*c-JUN*-flag cloning)

	ATGACG	
c-JUN-PR2	GTCCATGAATTCCGCGGATCCAAATGTTTGCAACTGCTGCGTTAGCATGAGTTGGCA	Human *c-JUN* rev (PB-CAG-*c-JUN*-flag cloning)
MBD3-PF	CTTCTTTTTCCTACAGCTCGAGATGGAGCGGAAGAGGTGGGAGTGCC	Human *MBD3* fwd (PB-CAG-*MBD3*-HA cloning)
MBD3-PR	GCCTCCGCTGCCTCCGGATCCGACGTGCTCCATCTCCGGGTCCG	Human *MBD3* rev (PB-CAG-*MBD3*-HA cloning)
MTA2-PF	CTTCTTTTTCCTACAGCTCGAGATGGAGATGAAGGTCTGGGACCCAGACAACCCTCTC	Human *MTA2* fwd (PB-CAG-*MTA2*-HA cloning)
MTA2-PR	AGCCTCCGCTGCCTCCGGATCCGTCCTCCAGGACAATAGGCTCATTGGTGCTGGCAG	Human *MTA2* rev (PB-CAG-*MTA2*-HA cloning)
MTA3-PF	CTTCTTTTTCCTACAGCTCGAGATGCTCGCAGATAAGCATGCTAAAGAAATTGAGG	Human *MTA3* fwd (PB-CAG-*MTA3*-HA cloning)
MTA3-PR	AGCCTCCGCTGCCTCCGGATCCGTCTGACACACAGCACGTGAGTTCATCCAGAC	Human *MTA3* rev (PB-CAG-*MTA3*-HA cloning)
**sgRNA sequence for knock-out human *c-JUN***		
**ID**	**Sequence (5'-3')**	**Description**
sgRNA1	ACAAGTTTCGGGGCCGCAAC	Human *c-JUN* knock-out sgRNA1 (Left sgRNA)
sgRNA2	GAGAACTTGACAAGTTGCGA	Human *c-JUN* knock-out sgRNA2 (Right sgRNA)
**shRNA sequence for knock-down human *MBD3***		

ID	Sequence (5’-3’)	Description
shRNA1-PF	CCGGGCCGGTGACCAAGATTACCA ACTCGAGTTGGTAATCTTGGTCACC GGCTTTTTG	Human *MBD3* knock down shRNA1
shRNA1-PR	AATTCAAAAAGCCGGTGACCAAGA TTACCAACTCGAGTTGGTAATCTTG GTCACCGGC	Human *MBD3* knock down shRNA1
shRNA2-PF	CCGGGACCTGAGCACCTTCGACTTC CTCGAGGAAGTCGAAGGTGCTCAG GTCTTTTTG	Human *MBD3* knock down shRNA2
shRNA2-PR	AATTCAAAAAGACCTGAGCACCTTC GACTTCCTCGAGGAAGTCGAAGGT GCTCAGGTC	Human *MBD3* knock down shRNA2

Antibodies

The following antibodies were used in this project:

Anti-FLAG (Sigma, Cat# F1804)

Anti-β-Catenin (Abcam, Cat# ab32572)

Anti-HA tag (CST, Cat# 3724s)

Anti-OCT4 (Abcam, Cat# ab19857)

Anti-SOX2 (Abcam, Cat# ab97959)

Anti-PDGFRA (Affinity, Cat# AF0241)

Anti-HAND1 Alexa Fluor® 647 (Novus, Cat# NBP2-71459AF647)

Anti-CD326 (EpCAM) Alexa Fluor® 488 (BioLegend, Cat# 324210)

Anti-c-JUN (CST, Cat# 9165)

Anti-EOMES (Affinity, Cat# DF8543)

Anti-GATA4 (Abcam, Cat# ab307823)

Anti-MBD3(Abcam, Cat# ab157464)

Anti-Histone H3 (Abcam, Cat# ab1791)

Anti-H3K4me3 (Abcam, Cat# ab8580)

Anti-S100 alpha 6/PRA antibody (Abcam, Cat# ab181975)

Alexa Fluor 488 goat anti-rabbit IgG (Invitrogen, Cat# A11008)

Anti-GAPDH HRP Conjugate (CST, Cat# 8884)

Anti-β-Actin HRP Conjugate (CST, Cat# 5125)

Anti-Rabbit IgG antibody (Abcam, Cat# ab6702)

Anti-Mouse IgG antibody (Abcam, Cat# ab6708)

Goat anti-Rabbit IgG (H+L) Secondary antibody HRP (Thermo Fisher Scientific, Cat# 31460)

Goat anti-Mouse IgG (H+L) Secondary antibody HRP (Thermo Fisher Scientific, Cat# 31430)

All antibodies used in this study are commercially available. The validation is available on the vendors website

Anti-FLAG: https://www.sigmaaldrich.com/MO/en/product/sigma/f1804

Anti-β-Catenin: https://www.abcam.com/products/primary-antibodies/beta-catenin-antibody-e247-chip-grade-ab32572.html

Anti-HA tag: https://www.cellsignal.com/products/primary-antibodies/ha-tag-c29f4-rabbit-mab/3724

Anti-OCT4: https://www.abcam.com/products/primary-antibodies/oct4-antibody-ab19857.html

Anti-SOX2: https://www.abcam.com/products/primary-antibodies/sox2-antibody-ab97959.html

Anti-PDGFRA: https://www.affbiotech.com/goods-157-AF0241-PDGF_Receptor_alpha_Antibody.html

Anti-HAND1 Alexa Fluor® 647: https://www.novusbio.com/products/hand1-antibody-oti1g10_nbp2-71459af647

Anti-CD326 (EpCAM) Alexa Fluor® 488: https://www.biolegend.com/en-gb/products/alexa-fluor-488-anti-human-cd326-epcam-antibody-3759?GroupID=BLG5134

Anti-c-JUN: https://www.cellsignal.com/products/primary-antibodies/c-jun-60a8-rabbit-mab/9165

Anti-EOMES: https://www.affbiotech.com/goods-12016-DF8543-EOMES_Antibody.html

Anti-GATA4: https://www.abcam.com/products/primary-antibodies/gata4-antibody-epr26718-103-ab307823.html

Anti-MBD3: https://www.abcam.com/products/primary-antibodies/mbd3-antibody-epr9913-chip-grade-ab157464.html

Anti-Histone H3: https://www.abcam.com/products/primary-antibodies/histone-h3-antibody-nuclear-marker-and-chip-grade-ab1791.html

Anti-H3K4me3: https://www.abcam.com/products/primary-antibodies/histone-h3-tri-methyl-k4-antibody-chip-grade-ab8580.html

Anti-S100 alpha 6/PRA antibody: https://www.abcam.com/products/primary-antibodies/s100-alpha-6pra-antibody-epr13084-69-ab181975.html

Alexa Fluor 488 goat anti-rabbit IgG: https://www.thermofisher.com/antibody/product/Goat-anti-Rabbit-IgG-H-L-Cross-Adsorbed-Secondary-Antibody-Polyclonal/A-11008

Anti-GAPDH HRP Conjugate: https://www.cellsignal.com/products/antibody-conjugates/gapdh-d16h11-xp-rabbit-mab-hrp-conjugate/8884

Anti-β-Actin HRP Conjugate: https://www.cellsignal.com/products/antibody-conjugates/b-actin-13e5-rabbit-mab-hrp-conjugate/5125

Anti-Rabbit IgG antibody: https://www.abcam.com/products/secondary-antibodies/goat-rabbit-igg-hl-ab6702.html

Anti-Mouse IgG antibody: https://www.abcam.com/products/secondary-antibodies/goat-mouse-igg-hl-ab6708.html

Goat anti-Rabbit IgG (H+L) Secondary Antibody HRP: https://www.thermofisher.com/antibody/product/Goat-anti-Rabbit-IgG-H-L-Secondary-Antibody-Polyclonal/31460

Goat anti-Mouse IgG (H+L) Secondary antibody HRP: https://www.thermofisher.com/antibody/product/Goat-anti-Mouse-IgG-H-L-Secondary-Antibody-Polyclonal/31430
